# Development of Ruminal and Fecal Microbiomes Are Affected by Weaning But Not Weaning Strategy in Dairy Calves

**DOI:** 10.3389/fmicb.2016.00582

**Published:** 2016-05-03

**Authors:** Sarah J. Meale, Shucong Li, Paula Azevedo, Hooman Derakhshani, Jan C. Plaizier, Ehsan Khafipour, Michael A. Steele

**Affiliations:** ^1^Department of Agricultural, Food and Nutritional Science, University of Alberta, EdmontonAB, Canada; ^2^Department of Animal Science, University of Manitoba, WinnipegMB, Canada; ^3^Department of Medical Microbiology, University of Manitoba, WinnipegMB, Canada; ^4^Nutreco Canada Inc., GuelphON, Canada

**Keywords:** 16S rRNA gene sequencing, microbiome, rumen, feces, Holstein dairy calves, MiSeq Illumina sequencing, weaning

## Abstract

The nature of weaning, considered the most stressful and significant transition experienced by dairy calves, influences the ability of a calf to adapt to the dramatic dietary shift, and thus, can influence the severity of production losses through the weaning transition. However, the effects of various feeding strategies on the development of rumen or fecal microbiota across weaning are yet to be examined. Here we characterized the pre- and post-weaning ruminal and fecal microbiomes of Holstein dairy calves exposed to two different weaning strategies, gradual (step-down) or abrupt. We describe the shifts toward a mature ruminant state, a transition which is hastened by the introduction of the solid feeds initiating ruminal fermentation. Additionally, we discuss the predicted functional roles of these communities, which also appear to represent that of the mature gastrointestinal system prior to weaning, suggesting functional maturity. This assumed state of readiness also appeared to negate the effects of weaning strategy on ruminal and fecal microbiomes and therefore, we conclude that the shift in gastrointestinal microbiota may not account for the declines in gain and intakes observed in calves during an abrupt weaning.

## Introduction

The ability of the ruminant forestomachs to convert fibrous feedstuff unsuitable for human consumption into volatile fatty acids (VFAs) and microbial proteins that can be utilized by the host to produce high quality protein in milk is of increasing importance in today’s society. Dynamic balances exist between ruminal and intestinal microbiomes, host physiology and diet that directly influence the initial acquisition, developmental succession and eventual stability of the rumen and intestinal ecosystems (Jami et al., 2013). Hence, an increased understanding of the factors affecting ruminal and intestinal development are crucial in optimizing dairy cow health and production efficiency.

During and immediately after birth, microbes from the cow and surrounding environment colonize the gastrointestinal (GI) tract of the calf, leading to the development of a complex microbiota (Bryant et al., 1958; Fonty et al., 1987; Minato et al., 1992). A number of factors may influence the process of gut microbial colonization in a calf including the cow’s vaginal microbiome and the proximity of the birth canal and the anus. Colostrum and milk microbiota may also influence the ecology of the rumen, although their impact on rumen development is expected to be little as they are shunted from the esophageal antrum to the abomasum through the esophageal groove (Church, 1976) in order to avoid wasteful microbial fermentation of milk in the rumen of sucking calves (Black and Sharkey, 1970). As such, extensive microbial fermentation in the rumen is considered to start following the introduction of solid feed (Hungate, 1966; Biesheuvel et al., 1991). Although, temporal shifts in the rumen microbiome with increasing age have been reported recently (Li et al., 2012; Jami et al., 2013; Rey et al., 2013), no study has characterized the stressful transition of the rumen microbiome from pseudo-monogastric to mature ruminant post-weaning. Furthermore, the implications of the weaning transition on the intestinal microbiome are also largely unknown.

The recent shift toward feeding elevated planes of nutrition to pre-weaned dairy calves to improve growth rates, feed efficiency and lifetime production (Drackley et al., 2007; Khan et al., 2011; Soberon and Van Amburgh, 2013) has prompted an examination of the effects of weaning strategy (gradual vs. abrupt weaning), as the stress of abruptly weaning calves off of a high plane of nutrition results in decreased intake and a loss in average daily gain (ADG) during the weaning period (Khan et al., 2007, 2011; Sweeney et al., 2010). There is, however, no information regarding the different effects of these two weaning strategies on the development of ruminal and intestinal microbiomes. It could be hypothesized that the decline in intake observed due to an abrupt weaning reflects the stress placed on the microbiome to immediately adapt to increased dietary concentrates. This is in contrast to a gradual weaning protocol, which has been suggested to increase starter intake and thus, facilitate a more gradual adaptation of the rumen microbiome minimizing negative effects on production. We hypothesized that the rumen microbiome would inevitably shift as a result of weaning and that the increased stress associated with an abrupt weaning would further alter the rumen microbiome from that observed in calves weaned gradually. As such, the objectives of this study were to characterize the shift in ruminal and intestinal microbiomes (the latter represented by fecal microbiome; Eckburg et al., 2005; Santiago et al., 2014) of dairy calves occurring as a result of weaning and the changes that occur in community structure as a result of different weaning strategies in Holstein dairy calves.

## Materials and Methods

### Animal Experiment and Sample Collection

This study was carried out in accordance with the recommendations of Nutreco Canada Agresearch Animal Care Committee in accordance with the Canadian Council on Animal Care (1993) guidelines. A detailed description of the experimental treatments and growth measurements was provided elsewhere (Steele et al., 2015). Briefly, 44 male and female Holstein dairy calves were blocked by gender and birthweight and randomly assigned to one of two weaning protocols: (1) Abrupt (control) or (2) Gradual (step-down) weaning. Both feeding regimes consisted of gradually increasing milk replacer by 0.5 L/d throughout the first week until calves were fed 9 L/d (4.5 L at 0600 and 1600 h). The abrupt weaning group was fed milk-replacer at 9 L/d until day 48 of life when milk replacer levels were reduced to 0 L within 24 h. The step-down weaning group was fed 9 L/d until day 36 when milk replacer levels were reduced to 4.5 L/d until day 48 (only 1600 h feeding) and then to 0 L on day 49. Calves had *ad libitum* access to water and starter (Optivia Advantage Calf Starter; 22% crude protein, CP) and chopped straw (8% CP) from days 7 to 54.

Rumen and fecal samples were collected on day 36 (pre-weaning) and day 54 (post-weaning) of life. Rumen fluid was collected at 1000 h using a modified Geishauser oral probe (1.3 cm diameter; Geishauser, 1993; Eckert et al., 2015). The probe was inserted 50 cm inside the calf and pH measurements of all samples were above 5.0, indicative of the rumen not the abomasum. Samples were collected in 15 mL tubes, flash frozen in liquid nitrogen and stored at -80°C. Calves were rectally finger-stimulated with sterile-gloved hand to facilitate the collection of a 50 g fecal sample, which was immediately frozen at -80°C.

Calves body weight (BW), starter intake, forage intake, fecal starch concentrations, rumen acetate, butyrate and propionate, and total rumen VFAs concentrations reported previously (Steele et al., 2015) were reinterpreted in the context of their correlations with microbial changes evaluated in this research.

### DNA Extraction

Rumen fluid and fecal samples were thawed at room temperature and kept on ice during the extraction process. Two hundred mg fecal samples or the sediment collected from 1 mL rumen fluid by centrifuging at 15,000 × *g* for 5 min was used for DNA extraction using a ZR fecal DNA kit (D6010; Zymo Research Corp., Irvine, CA, USA) that included a bead-beating step for mechanical disruption of microbial cells. DNA was eluted from the column with elution buffer, and DNA concentration was quantified using a NanoDrop 2000 spectrophotometer (Thermo Scientific, Waltham, MA, USA). DNA samples were normalized to 50 ng/μL, and quality verified by PCR amplification of the 16S rRNA gene using universal primers 27F (5′-GAAGAGTTTGATCATGGCTCAG-3′) and 342R (5′-CTGCTGCCTCCCGTAG-3′), as described before (Khafipour et al., 2009). Amplicons were verified by agarose gel electrophoresis. All DNA samples were stored at -80°C.

### Library Construction and Illumina Sequencing

The bacterial V4 region of 16S rRNA gene was targeted for PCR amplification using modified F515/R806 primers (Caporaso et al., 2012) as described before (Derakhshani et al., 2016). The reverse PCR primer was indexed with 12-base Golay barcodes allowing for multiplexing of samples. PCR reaction for each sample was performed in duplicate and contained 1.0 μL of pre-normalized DNA, 1.0 μL of each forward and reverse primers (10 μM), 12 μL HPLC grade water (Fisher Scientific, Ottawa, ON, Canada) and 10 μL 5 Prime Hot MasterMix (5 Prime, Inc., Gaithersburg, MD, USA). Reactions consisted of an initial denaturing step at 94°C for 3 min followed by 35 amplification cycles at 94°C for 45 s, 50°C for 60 s, and 72°C for 90 s; finalized by an extension step at 72°C for 10 min in an Eppendorf Mastercycler pro (Eppendorf, Hamburg, Germany). PCR products were then purified using ZR-96 DNA Clean-up Kit (ZYMO Research, Irvine, CA, USA) to remove primers, dNTPs and reaction components. The V4 library was generated by pooling 200 ng of each sample, quantified by Picogreen dsDNA (Invitrogen, Grand Island, NY, USA). This was followed by multiple dilution steps using pre-chilled hybridization buffer (HT1; Illumina, San Diego, CA, USA) to bring the pooled amplicons to a final concentration of 5 pM, measured by Qubit 2.0 Fluorometer (Life Technologies, Burlington, ON, Canada). Finally, 15% of PhiX control library was spiked into the amplicon pool to improve the unbalanced and biased base composition, a known characteristic of low diversity 16S rRNA libraries. Customized sequencing primers for read1 (5′-TATGGTAATTGTGTGCCAGCMGCCGCGGTAA-3′), read2 (5′-AGTCAGTCAGCCGGACTACHVGGGTWTCTAAT-3′) and index read (5′-ATTAGAWACCCBDGTAGTCCGGCTGACTGACT-3′) were synthesized and purified by polyacrylamide gel electrophoresis (Integrated DNA Technologies, Coralville, IA, USA) and added to the MiSeq Reagent Kit V2 (300-cycle; Illumina, San Diego, CA, USA). The 150 paired-end sequencing reaction was performed on a MiSeq platform (Illumina, San Diego, CA, USA) at the Gut Microbiome Laboratory (Department of Animal Science, University of Manitoba, Winnipeg, MB, Canada). The sequencing data were deposited into the Sequence Read Archive (SRA) of NCBI^1^ and can be accessed via accession number SRR3031098.

### Bioinformatic Analysis

The PANDAseq assembler (Masella et al., 2012) was used to merge and fix the overlapping paired-end Illumina fastq files. All the sequences with low quality base calling scores, as well as those containing uncalled bases (N) in the overlapping region were discarded. The output fastq file was then analyzed by downstream computational pipelines of the open source software package QIIME (Caporaso et al., 2010b). The default minimum quality threshold of 25 was used. Chimeric sequences were detected using the UCHIME algorithm (USEARCH 6.1) to run both *de novo* and reference based chimera detection. The number of chimeric sequences identified and consequently removed by both detection methods was 1.7% of total high quality sequences. Sequences were clustered at the 97% sequence similarity level using the Greengenes database (Version 13.5; Edgar et al., 2011) using an open reference-based OTU picking approach with the QIIME algorithm and usearch61 method with default parameters (Edgar et al., 2011; McDonald et al., 2012). Those sequences that failed to cluster were subsampled for *de novo* OTU picking. All picked OTUs were subsequently aligned by PyNAST (Caporaso et al., 2010a) and a phylogenetic tree was built using FastTree method (Price et al., 2009) to calculate UniFrac distances (Lozupone et al., 2011) within QIIME. Representative OTUs were assigned to bacterial taxonomies using RDP classifier via QIIME with a confidence threshold of 0.8 (Wang et al., 2007). Finally, open source software PICRUSt (phylogenetic investigation of communities by reconstruction of unobserved states; Langille et al., 2013) was used on 16S rRNA gene sequencing data to predict functional genes of the classified members of the rumen and fecal microbiota resulting from reference-based OTU picking against Greengenes database. Predicted genes were then hierarchically clustered and categorized under Kyoto Encyclopedia of Genes and Genomes (KEGG; Kanehisa and Goto, 2000) orthologs (KOs) and pathways (level 3; Supplementary Tables 3 and 4).

### Alpha- and Beta-Diversity Analyses

The phylogenetic tree was rooted using *Methanococcus jannaschii* (L77117) as an outgroup. Subsequently an OTU table was generated by QIIME, which along with the mapping file was assembled into a Phyloseq object (McMurdie and Holmes, 2013). For within community diversity (α-diversity) calculations, the same number of sequences for each sample were randomly selected [corresponding to the sample with lowest number of sequences (see Results), one sample had only 129 sequences and was discarded], in order to eliminate the bias caused by different sample sizes (Roesch et al., 2007). Alpha-diversity analysis was conducted with standard diversity metrics accessed via Phyloseq, including observed richness, Chao1, Shannon index, Simpson index, Inverse Simpson index, and Fisher index. The dataset was also subsampled to the minimum (Carcer et al., 2011) to compare microbial compositions between samples (β-diversity). Beta-diversity was measured by calculating the weighted and unweighted UniFrac distances (Lozupone and Knight, 2005) using Phyloseq default scripts. Principal coordinate analysis (PCoA) was applied on the resulting distance matrices to generate two-dimensional plots using PRIMER v6 software (Warwick and Clarke, 2006). Permutational multivariate analysis of variance (PERMANOVA; Anderson, 2005) was used to calculate *P*-values and to test differences of β-diversity among treatment groups for significance. Both weighted and unweighted UniFrac distances were used to compute the test statistic and *P*-values (Kuczynski et al., 2010).

### Bacterial Community Composition and Metagenome prediction

Bacterial community composition at the phylum and genus levels, and predictive metagenome profiles were compared among treatments (abrupt vs. gradual weaning) and days (pre- vs. post-weaning) using the nbinomWaldTest method of DESeq2 (Love et al., 2014) according to a randomized design with pre-weaning as covariate and containing dietary treatment, day and calf; these were considered significant at *P* < 0.05 (DESeq2, R package version 1.8.1, 2014).

### Correlations between Metabolites and Bacterial Abundance

Non-parametric Spearman rank correlation coefficient analysis implemented in PAST software (Hammer et al., 2012) was used to test the relationship between BW, starter intake, forage intake, fecal starch, molar proportions of rumen acetate, rumen butyrate, rumen propionate, total rumen VFA concentration and the bacterial communities in rumen fluid and feces. The resulting correlation matrix was visualized in a heatmap format generated by the corrplot package of R (Corrplot: visualization of a correlation matrix; R package version 0.73. 2013^2^).

## Results

### Alpha-Diversity Measures

For rumen samples, the lowest number of sequences per sample was 28,463. Whereas, for fecal samples, the lowest number of sequences per sample was 16,711. Rumen microbiota were more diverse and had greater evenness in pre-weaned calves, compared to ruminal microbiota in post-weaned calves, as indicated by Chao1, ACE, Shannon and Simpson indices (*P* < 0.05; **Table 1**). Conversely, fecal microbiota had a greater richness and evenness post-weaning compared to pre-weaning (*P* < 0.05; **Table 1**). No effects of weaning strategy were observed on microbiota richness or diversity in the rumen or feces.

**Table 1:** Alpha diversity indices (mean ± standard error).

	Pre-weaning	Post-weaning	*P*-value^1^
**Rumen**			
Observed	1903 ± 69	1561 ± 68	<0.001
Chao1	2357 ± 137	2010 ± 136	0.02
ACE	2546 ± 146	2186 ± 144	0.02
Shannon	4.327 ± 0.076	3.917 ± 0.076	<0.001
Simpson	0.932 ± 0.008	0.905 ± 0.008	0.02
InvSimpson	20.27 ± 1.37	13.47 ± 1.36	<0.001
Fisher	425 ± 18	330 ± 18	<0.001
**Feces**			
Observed	1774 ± 81	2556 ± 81	<0.001
Chao1	2452 ± 194	3662 ± 194	<0.001
ACE	2661 ± 206	4042 ± 206	<0.001
Shannon	4.401 ± 0.066	5.038 ± 0.066	<0.001
Simpson	0.936 ± 0.004	0.964 ± 0.004	<0.001
InvSimpson	21.52 ± 2.24	38.20 ± 2.24	<0.001
Fisher	398 ± 22	612 ± 22	<0.001

*^1^P-values for treatment effect (weaning strategy) were never significant and are thus, not presented.*

### OTU Diversity and Similarity Analysis

**FIGURE 1:**
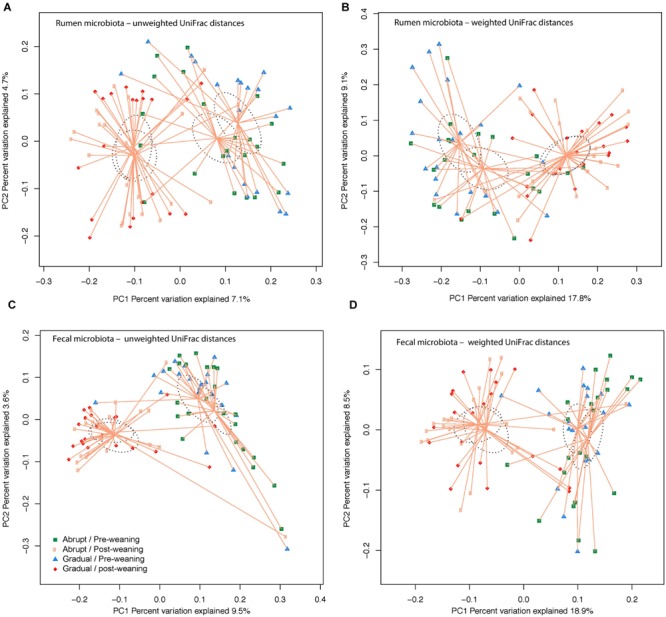
**Similarity of the bacterial communities between weaning strategy (abrupt vs. gradual weaning; *P* > 0.05) and weaning status (pre- vs. post-weaning; *P* < 0.001).** Distance between the samples, based on similarity in OTU composition (OTU similarity ≥ 97%) calculated using **(A)** unweighted UniFrac distances in the rumen; **(B)** weighted UniFrac distance in the rumen; **(C)** unweighted UniFrac distance in feces; and **(D)** weighted UniFrac distance in feces, and were visualized in principal coordinates analysis (PCoA) plots. A greater distance between two points infers a lower similarity, whereas similar OTUs cluster together. The impact of weaning and weaning strategy on the clustering pattern of microbial communities were tested using PERMANOVA (implemented in PRIMER-6 software). *P* < 0.05 were considered significant.

Community OTU comparisons by PCoA analysis (OTU ≥ 97% identity, species level similarity) using weighted and unweighted UniFrac distance similarity metrics revealed that both rumen and fecal samples were clustered according to pre- and post-weaning status (days 36 and 54 of life; *P* < 0.001; **Figure 1**). Yet, weaning strategy did not affect clustering (*P* > 0.05; **Figure 1**), suggesting that post-weaned calves have distinct ruminal and fecal microbial communities compared to pre-weaned calves, regardless of their weaning strategy.

### Bacterial Composition of Rumen and Fecal Microbiota

**FIGURE 2:**
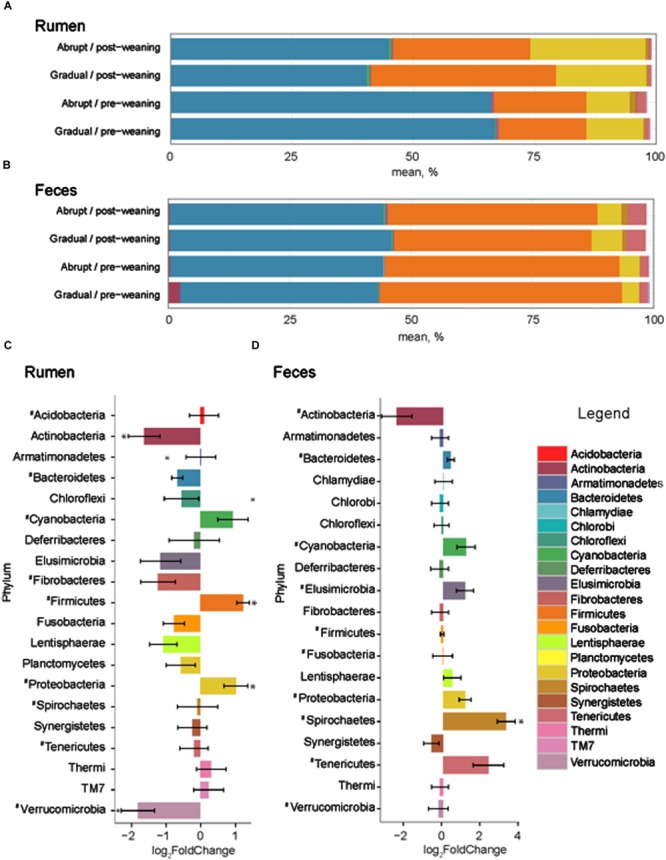
**Phylum level composition.** Color-coded bar plots showing average bacterial phyla distribution (%) in **(A)** rumen and **(B)** feces of pre- and post-weaned calves for each weaning strategy. Average log_2_ fold change comparisons between pre- and post-weaned calves across both weaning strategies are also presented in the **(C)** rumen and **(D)** feces. ^∗^ Represents significant differences (*P* < 0.05) between pre- vs. post-weaned calves. # Represents taxa present at >0.1% of the community.

The number of input 16S rRNA sequence reads generated from rumen and fecal microbiota were 44,008 and 44,050, respectively. While the majority of OTUs were identified at the genus (g.) or species levels, some were only classified at the phylum (p.), class (c.), order (o.), or family (f.) level. Phylogenetic analysis of these sequences identified 21 phyla from the rumen, 12 of which had a relative abundance > 0.1% of the community. The three most abundant phyla in the rumen were Bacteroidetes, Firmicutes and Proteobacteria (**Figure 2**). These phyla accounted for an average of 96.1% of the community, regardless of weaning status or weaning strategy, where the relative abundance of p. Bacteroidetes decreased (*P* = 0.03) from 66.1 to 42.2% in pre- vs. post-weaned calves (**Figure 2**). This was compensated for by increases (*P* ≤ 0.04) in Proteobacteria (10.5–20.3%) and Firmicutes (18.6–34.4%) during the weaning transition. Other phyla affected (*P* ≤ 0.002) by weaning included Actinobacteria and Verrucomicrobia, the proportion of which decreased in the more mature weaned rumen (**Figure 2**).

Of the 315 genera observed in the rumen, 67 had a relative abundance over 0.1%, and changed between pre- and post-weaning (**Table 2**). The abundance of the dominant g. *Prevotella* remained stable in the rumen despite weaning or weaning strategy, accounting for an average of 36.3 and 32.7%, in pre- and post-weaned calves, respectively (Supplementary Table 1). Conversely, g. *Succinivibrio* was the most abundant genus in p. Proteobacteria of pre-weaned calves but declined following weaning (2.12 vs. 0.71%, respectively; *P* = 0.004). In the p. Firmicutes, *Sharpea* was the dominant genus in post-weaned calves increasing from 1.48 to 7.67% as a result of weaning (*P* < 0.001).

**Table 2:** Mean abundances of bacterial taxa present at >0.1% of total ruminal sequences which were significantly different (*P* < 0.05) between pre- vs. post-weaned calves^1^.

Phylum	Taxa^2^	Pre-weaning	Post-weaning	log_2_ FC	*SE*^3^	*P*-value^4^
Actinobacteria	g. *Bifidobacterium*	0.080	0.085	-2.24	0.594	<0.001
Bacteroidetes	f. [Barnesiellaceae]	0.066	0.002	-3.43	0.722	<0.001
	g. *Butyricimonas*	0.647	0.051	-4.12	0.411	<0.001
	g. *Odoribacter*	0.078	0.004	-4.44	0.448	<0.001
	g. *CF231*	0.896	0.235	-2.39	0.357	<0.001
	f. [Paraprevotellaceae]	0.421	0.861	0.65	0.227	0.009
	g. *Bacteroides*	3.62	0.863	-2.43	0.305	<0.001
	g. *BF311*	0.063	0.006	-2.47	0.857	0.008
	f. Bacteroidaceae	0.046	0.005	-4.53	0.541	<0.001
	f. BS11	1.57	0.045	-1.76	0.653	0.013
	o. Bacteroidales	16.5	6.07	-1.47	0.211	<0.001
	f. p-2534-18B5	0.341	0.056	-3.43	0.813	<0.001
	g. *Parabacteroides*	0.435	0.020	-4.51	0.402	<0.001
	f. Prevotellaceae	0.124	0.051	-0.99	0.338	0.007
	f. Rikenellaceae	0.207	0.009	-5.02	0.515	<0.001
	g. *Sphingobacterium*	0.009	0.002	-2.14	0.676	0.004
Cyanobacteria	o. Streptophyta	0.001	0.010	3.74	0.604	<0.001
Elusimicrobia	g. *Elusimicrobium*	0.112	0.002	-4.64	0.886	<0.001
	f. Elusimicrobiaceae	0.064	0.003	-2.72	0.741	<0.001
Fibrobacteres	g. *Fibrobacter*	0.264	0.123	-1.48	0.540	0.012
Firmicutes	f. Lactobacillaceae	0.008	0.031	2.66	0.514	<0.001
	f. Streptococcaceae	0.089	0.003	-3.77	0.607	<0.001
	g. *Streptococcus*	0.347	0.004	-3.16	0.558	<0.001
	f. Christensenellaceae	0.229	0.020	-3.18	0.580	<0.001
	g. *Clostridium*	0.064	0.003	-4.17	0.811	<0.001
	g. *Pseudoramibacter*	0.006	0.075	3.12	1.026	0.005
	g. *Butyrivibrio*	1.27	3.39	1.41	0.207	<0.001
	g. *Coprococcus*	0.485	0.229	-1.00	0.333	0.006
	f. Lachnospiraceae	1.60	2.54	0.89	0.180	<0.001
	g. *Pseudobutyrivibrio*	0.011	0.005	-1.99	0.682	0.007
	g. *Shuttleworthia*	0.477	1.68	4.22	0.403	<0.001
	o. Clostridiales	1.39	1.35	-0.51	0.230	0.048
	g. *Anaerotruncus*	0.008	0.004	-2.40	0.726	0.002
	f. Ruminococcaceae	2.45	5.50	1.35	0.199	<0.001
	g. *Oscillospira*	0.819	0.251	-1.84	0.234	<0.001
	g. *Ruminococcus*	1.27	1.25	-1.50	0.284	<0.001
	g. *Acidaminococcus*	0.076	0.118	3.77	0.540	<0.001
	g. *Dialister*	0.158	1.33	4.34	0.404	<0.001
	g. *Megasphaera*	0.452	0.634	2.34	0.394	<0.001
	g. *Mitsuokella*	0.013	0.022	1.78	0.507	0.001
	f. Veillonellaceae	0.557	1.59	2.28	0.327	<0.001
	g. *Succiniclasticum*	0.365	0.218	-1.69	0.444	<0.001
	o. Coriobacteriales	0.158	0.006	-3.94	0.522	<0.001
	f. [Coprobacillaceae]	0.002	0.004	2.21	0.792	0.011
	g. *Sharpea*	1.48	7.67	3.81	0.423	<0.001
	g. *[Eubacterium]*	0.009	0.053	3.28	0.625	<0.001
	g. *Bulleidia*	0.368	0.487	1.60	0.348	<0.001
	g. *L7A_E11*	0.022	0.003	-2.57	0.557	<0.001
	g. *p-75-a5*	0.056	0.012	-1.88	0.468	<0.001
	o. ML615J-28	0.033	0.002	-2.14	0.868	0.024
Lentisphaerae	f. Victivallaceae	0.007	0.001	-2.99	1.049	0.009
Proteobacteria	f. Alcaligenaceae	0.043	0.010	-2.57	0.680	<0.001
	g. *Sutterella*	0.094	0.015	-1.22	0.449	0.013
	f. Comamonadaceae	0.320	0.066	-1.61	0.535	0.006
	c. Betaproteobacteria	0.281	0.021	-3.38	0.391	<0.001
	f. Neisseriaceae	0.012	0.005	-1.61	0.617	0.017
	g. *Desulfovibrio*	0.473	0.649	0.72	0.261	0.012
	g. *Campylobacter*	0.178	0.060	-0.80	0.309	0.017
	f. Succinivibrionaceae	6.07	17.9	2.21	0.335	<0.001
	g. *Ruminobacter*	0.252	0.074	-4.19	0.840	<0.001
	g. *Succinivibrio*	2.12	0.712	-1.35	0.430	0.004
	c. Gammaproteobacteria	0.075	0.204	1.74	0.329	<0.001
	f. Pasteurellaceae	0.012	0.002	-2.25	0.521	<0.001
	g. *Acinetobacter*	0.045	0.027	-1.97	0.384	<0.001
	g. *Psychrobacter*	0.006	0.000	-2.88	0.815	0.001
Tenericutes	g. *RFN20*	0.913	0.091	-2.92	0.451	<0.001
Verrucomicrobia	f. RFP12	0.246	0.019	-2.88	0.522	<0.001
	f. WCHB1-25	0.009	0.002	-2.60	0.948	0.012

*^1^Bacterial taxa present in at least 50% of calves and represented at least 0.1% of the bacterial community in at least one calf. ^2^Sequences that could not be assigned to a genus are displayed using the highest taxonomic level (class, c.; order, o.; family, f.) they could be assigned to. ^3^FC, fold change; SE, standard error for the log_*2*_ fold change estimate.^4^*P*-values for treatment effect (weaning strategy) were never significant, and thus, are not presented. Ruminal taxa present but not significantly different between pre- and post-weaned calves are presented in Supplementary Table 1.*

**FIGURE 3:**
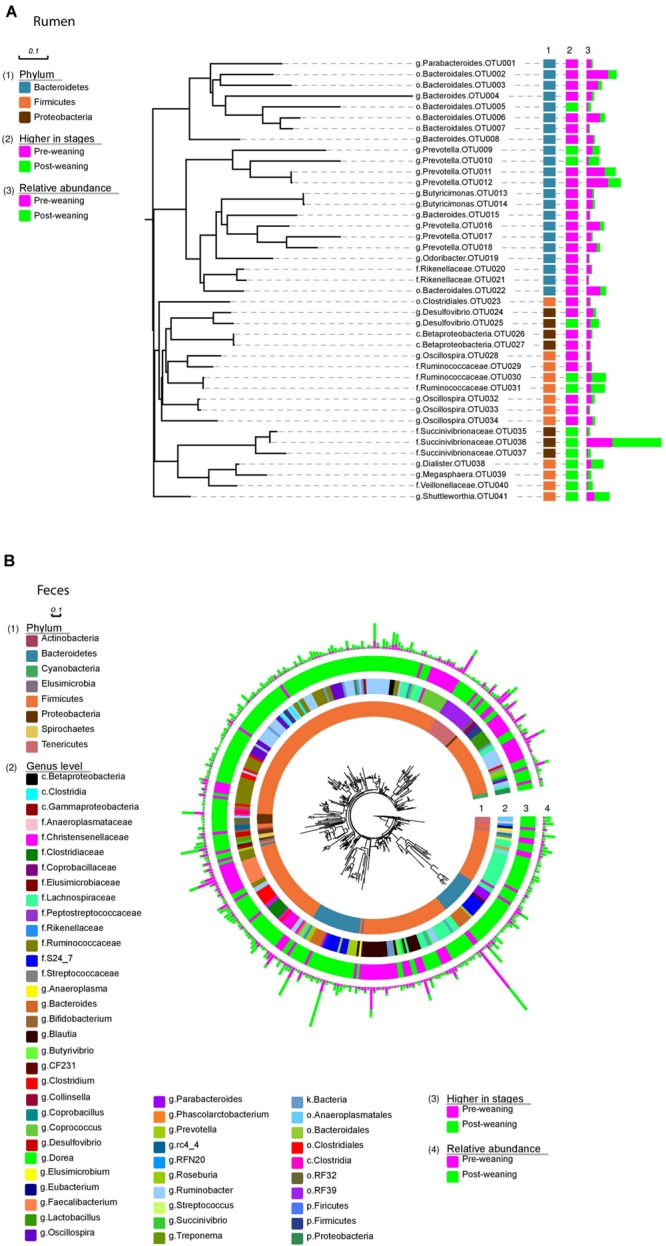
**Bacteria identified at OTU level.** Rumen bacteria **(A)** identified at OTU level arranged into phyla (1); changes in abundance between pre- and post-weaning (2); and the relative abundance of each OTU in both pre- and post-weaned calves (3). Fecal bacteria **(B)** identified at OTU level arranged into phyla (1); genus level (2); changes in abundance between pre- and post-weaning (3) and the relative abundance of each OTU in both pre- and post-weaned calves (4).

Twenty phyla were identified in the fecal microbiome, 10 of which had a relative abundance > 0.1% of the total community (**Figure 2**). However, similar to the rumen, the weaning strategy did not impact the relative abundance of microbiota of calves. The most abundant phyla in feces were also the same as those in the rumen; however, the dominant phylum did not differ between pre- and post-weaning. The weaning transition numerically shifted Firmicutes from the dominant pre-weaning phylum to the second most abundant phylum post-weaning, behind Bacteroidetes (49.1 vs. 42.3% pre-weaning; 41.8 vs. 44.8% post-weaning, respectively). Proteobacteria remained the third most abundant phylum with a relative abundance of 4.1% pre-weaning, compared to 5.4% post-weaning. Only p. Spirochaetes was significantly impacted by weaning in the fecal microbial community, increasing on average 3.29 times in weaned calves (*P* < 0.001; **Figure 3**).

Despite a decrease in its relative abundance (*P* < 0.001), *Bacteroides* remained the predominant genus in feces of pre- and post-weaned calves (30.98 and 17.78%, respectively; **Table 3**). In the feces of pre-weaned calves, the dominant genus in p. Firmicutes was *Blautia* (9.82%) which declined (*P* < 0.001) to 2.49% in post-weaned calves. The dominant genus within this phyla became *Ruminococcus* in weaned calves with an average relative post-weaning abundance of 10.41% (*P* < 0.001; **Table 3**). Similarly, *Escherichia* (1.21%; Supplementary Table 2) was the most abundant genus in p. Proteobacteria pre-weaning but showed no change between pre- and post-weaning, consequently, an increase (*P* < 0.001) in post-weaned calves lead to g. *Succinivibrio* becoming the dominant genera in this phylum in feces of weaned calves (2.29%; **Table 3**).

**Table 3:** Mean abundances of bacterial taxa present at >0.1% of total fecal sequences which were significantly different (*P* < 0.05) between pre- vs. post-weaned calves^1^.

Phylum	Taxa^2^	Pre-weaning	Post-weaning	log_2_ FC	*SE*^3^	*P*-value^4^
Actinobacteria	g. *Bifidobacterium*	1.21	0.341	-2.54	0.417	<0.001
Bacteroidetes	g. *CF231*	0.186	0.399	1.39	0.368	<0.001
	g. *Bacteroides*	31.0	17.8	-1.17	0.139	<0.001
	o. Bacteroidales	1.98	3.55	0.92	0.230	<0.001
	g. *Prevotella*	1.15	3.29	1.28	0.274	<0.001
	f. S24-7	1.46	10.9	3.53	0.278	<0.001
Elusimicrobia	g. Elusimicrobium	0.026	0.192	1.64	0.453	0.001
Firmicutes	g. *Lactobacillus*	2.87	0.195	-3.63	0.355	<0.001
	f. Lactobacillaceae	0.320	0.039	-2.94	0.398	<0.001
	o. Lactobacillales	0.008	0.001	-5.01	0.892	<0.001
	f. Streptococcaceae	1.55	0.271	-3.62	0.326	<0.001
	g. *Streptococcus*	0.174	0.026	-4.22	0.364	<0.001
	f. Christensenellaceae	0.101	0.488	2.39	0.377	<0.001
	g. *Clostridium*	0.094	0.246	2.36	0.447	<0.001
	f. Clostridiaceae	0.230	0.659	1.46	0.220	<0.001
	g. *Eubacterium*	0.007	0.002	-2.43	0.981	0.032
	g. *Blautia*	9.82	2.49	-2.68	0.250	<0.001
	g. *Butyrivibrio*	0.077	0.222	1.40	0.312	<0.001
	g. *Coprococcus*	3.74	0.675	-2.72	0.281	<0.001
	g. *Dorea*	0.801	0.334	-1.40	0.320	<0.001
	f. Lachnospiraceae	2.90	5.58	0.37	0.134	0.015
	g. *Roseburia*	0.055	0.347	2.56	0.315	<0.001
	o. Clostridiales	0.602	3.11	1.99	0.212	<0.001
	g. *rc4-4*	0.075	0.272	2.09	0.366	<0.001
	f. Peptostreptococcaceae	0.177	0.085	-1.30	0.370	0.001
	g. *Faecalibacterium*	4.07	0.944	-2.48	0.331	<0.001
	g. *Ruminococcus*	3.04	10.4	1.50	0.186	<0.001
	g. *Anaerovibrio*	0.098	0.364	1.19	0.348	0.002
	f. Veillonellaceae	0.037	0.088	0.96	0.302	0.004
	g. *Collinsella*	0.236	0.024	-3.13	0.387	<0.001
	g. *Eggerthella*	0.027	0.006	-2.50	0.701	0.001
	o. Coriobacteriales	0.005	0.018	1.37	0.413	0.003
	f. [Coprobacillaceae]	2.91	0.810	-3.39	0.336	<0.001
	g. *[Eubacterium]*	0.141	0.103	-1.89	0.322	<0.001
	g. *Bulleidia*	0.014	0.032	1.11	0.463	0.038
	f. Erysipelotrichaceae	0.038	0.265	1.04	0.346	0.007
Proteobacteria	o. RF32	0.151	0.314	0.96	0.365	0.022
	o. NA	0.694	1.02	1.04	0.306	0.002
	g. *Helicobacter*	0.017	0.010	-2.16	0.660	0.003
	g. *Succinivibrio*	0.477	2.29	1.55	0.382	<0.001
Spirochaetes	g. *Treponema*	0.057	1.157	3.08	0.363	<0.001
Tenericutes	f. Anaeroplasmataceae	0.084	1.33	3.77	0.419	<0.001
	o. Anaeroplasmatales	0.169	1.09	4.00	0.405	<0.001
	c. Mollicutes	0.007	0.013	1.45	0.563	0.025

*^1^Bacterial taxa present in at least 50% of calves and represented at least 0.1% of the bacterial community in at least one calf. ^2^Sequences that could not be assigned to a genus are displayed using the highest taxonomic level (class, c.; order, o.; family, f.) they could be assigned to. ^3^FC, fold change; SE, standard error for the log_*2*_ fold change estimate. ^4^*P*-values for treatment effect (weaning strategy) were never significant, and are thus, not presented. Fecal taxa present but not significantly different between pre- and post-weaned calves are presented in Supplementary Table 2.*

Of the 40 OTUs with a relative abundance > 0.1% of the total community, 13 increased and 28 decreased in the ruminal microbiota of weaned calves, compared to pre-weaning (**Figure 3A**). These 41 OTUs accounted for approximately 65% of all sequences and all belonged to the three dominant phyla, Bacteroidetes, Firmicutes and Proteobacteria. Comparatively, 235 OTUs with a relative abundance > 0.1% of the community were identified in the fecal microbiota of both pre- and post-weaned calves, and changed as a result of weaning (**Figure 3B**). These OTUs were classified as belonging to several other phyla (Actinobacteria, Cyanobacteria, Elusimicrobia, Spirochaetes, and Tenericutes) that were not found in the rumen.

### Correlation between Rumen and Fecal Microbiota and Calves Physiological Variables

**FIGURE 4:**
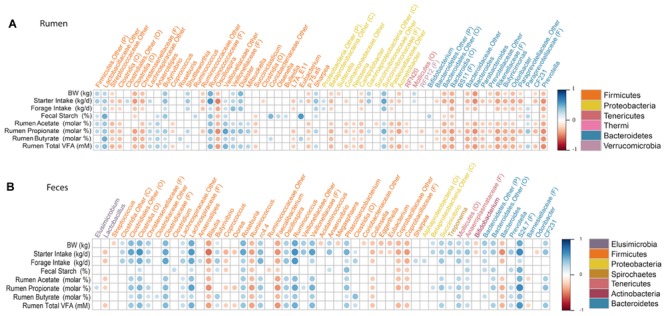
**Correlations between production variables and relative taxa abundance.** Spearman non-parametric rank correlation matrix of the dominant bacterial genera across **(A)** rumen and **(B)** fecal samples. The genera were included in the matrix if they were in at least 50% of calves and represented at least 0.1% of the bacterial community in at least one calf. Individual VFA represent molar proportions of each VFA, molar%. All correlations presented were statistically significant (*P* < 0.05), with strong correlations indicated by large circles and weaker correlations indicated by small circles. The scale colors denote whether the correlation is positive (closer to 1, blue circles) or negative (closer to -1, red circles) between the taxa and production variables.

Overall, 327 and 235 genera were detected in the rumen and feces. However, we only compared genera that were present in at least 50% of calves and represented at least 0.1% of the bacterial community in at least one calf, to the physiological variable. Our assumption was that these genera represent important components of the healthy rumen ecosystem, and would therefore, be more likely to reveal a connection between host physiology and the gastrointestinal bacterial community. A correlation matrix was created to evaluate the relative abundance of each of these genera with calves biophysical variables and rumen fermentation characteristics (**Figure 4**). The relative abundance of g. *Bacteroides* was negatively correlated (*P* ≤ 0.006) with BW (Spearman’ ρ = -0.45; Spearman’ ρ = -0.29), starter (Spearman’ ρ = -0.47; Spearman’ ρ = -0.47) and forage intakes (Spearman’ ρ = -0.32; Spearman’ ρ = -0.32), and ruminal concentration of acetate (Spearman’ ρ = -0.36; Spearman’ ρ = -0.26), propionate (Spearman’ ρ = -0.47; Spearman’ ρ = -0.37), butyrate (Spearman’ ρ = -0.35; Spearman’ ρ = -0.30) and total VFA (Spearman’ ρ = -0.44; Spearman’ ρ = -0.34) in the rumen and feces, respectively. In the rumen, the relative abundance of g. *Prevotella* was not correlated (*P* ≥ 0.216) to any production or intake traits; however, in the fecal microbiota, *Prevotella* was positively correlated (*P* ≤ 0.017) with starter intake (Spearman’ ρ = 0.25), ruminal propionate (Spearman’ ρ = 0.26) and butyrate concentrations (Spearman’ ρ = 0.25), as well as total VFA concentration (Spearman’ ρ = 0.36). The relative abundance of g. *Succinivibrio* was positively correlated (*P* = 0.011) with BW (Spearman’ ρ = 0.33), starter intake (Spearman’ ρ = 0.34) and ruminal concentrations of acetate (Spearman’ ρ = 0.27), propionate (Spearman’ ρ = 0.38) and total VFA (Spearman’ ρ = 0.32) in the feces. Whereas, g. *Sharpea* was positively correlated (*P* ≤ 0.030) to BW (Spearman’ ρ = 0.28), starter (Spearman’ ρ = 0.40) and forage intakes (Spearman’ ρ = 0.31), fecal starch (Spearman’ ρ = 0.23) and subsequent increases in ruminal concentration of propionate (Spearman’ ρ = 0.30) and total VFA (Spearman’ ρ = 0.26). Additionally, the relative abundance of g. *Ruminococcus* was strongly correlated (*P* < 0.0001) with BW (Spearman’ ρ = 0.51), starter (Spearman’ ρ = 0.60) and forage intakes (Spearman’ ρ = 0.57) in the feces.

### Functional and Metabolic Capacity of Ruminal and Fecal Microbiomes

**FIGURE 5:**
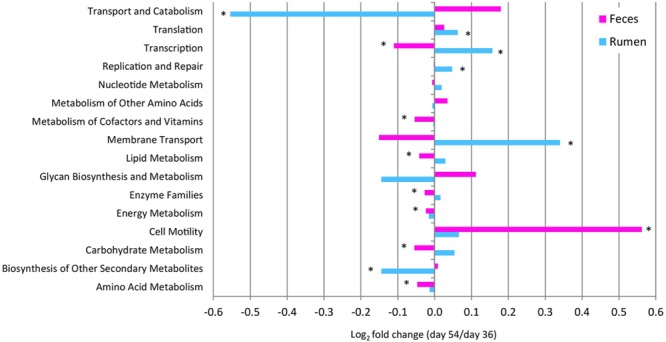
**Fold change in the number of 16S rRNA gene sequences annotated to KEGG level 2 orthologies representing the predicted functional composition of the rumen and feces of pre- vs. post-weaned calves.** ^∗^ Represents significant differences (*P* < 0.05) between pre- vs. post-weaned calves.

To assess the metabolic potentials of pre- vs. post-weaned ruminal and fecal microbiomes, OTUs were entered into PICRUSt and the inferred gene families were annotated against KOs. A total of 11 level 2 pathways from the rumen and 14 from the feces showed differing numbers of OTUs assigned to each pathway for pre- vs. post-weaned calves (**Figure 5**). Carbohydrate metabolism and lipid metabolism decreased (*P* ≤ 0.02) in feces (indicative of the intestine) of post- vs. pre-weaned calves. A corresponding increase in both carbohydrate metabolism and lipid metabolism of post-weaned calves was observed in the rumen; however, the differences were not significant. Similarly, energy metabolism and amino acid metabolism increased (*P* ≤ 0.01) in feces of post-weaned calves compared to pre-weaned calves. A total of 292 KO level 3 pathways were identified in the rumen and 286 from feces (Supplementary Tables 3 and 4). In the rumen and feces, the most abundant level 3 pathway was transporters (KO02000), regardless of weaning, accounting for ≥4.73% of all sequence reads. Other abundant KOs (with >2% sequence reads assigned) across all samples included ABC transporters (KO02010), ribosome (KO03010) and purine metabolism (KO00230). The number of OTUs assigned to KOs differed between pre- and post-weaned ruminal and fecal microbiomes for 59 and 64 of the pathways, respectively (*P* < 0.05; Supplementary Tables 3 and 4). Of the OTUs assigned to KOs in the rumen, only four with an abundance greater than 1% were related to carbohydrate metabolism, including amino sugar and nucleotide sugar metabolism (KO00520), glycolysis/gluconeogenesis (KO00010), fructose and mannose metabolism (KO00051) and starch and sucrose metabolism (KO00500). However, the number of OTUs assigned to each pathway was stable across weaning (*P* > 0.05; Supplementary Table 3). Comparatively, in the fecal microbiome, the number of OTUs assigned to the fructose and mannose metabolism pathways, as well as the starch and sucrose metabolism pathways decreased post-weaning (*P* < 0.001).

## Discussion

The progressive development of a calf’s ruminal and intestinal microbiomes begin at birth with the most significant change occurring at weaning. Initiating fermentative processes in the rumen greatly alters the microbial composition of the rumen and that of the lower intestinal tract. In this study, we characterized the shift in ruminal and intestinal microbiomes (as represented by the feces; Santiago et al., 2014) occurring as a result of weaning. Additionally, we determined if the weaning strategy influenced the development of microbiota post-weaning in the GI tract, as this may provide an explanation for the negative production effects observed during weaning. Collectively, our results suggest that despite being fed at an elevated plane of nutrition the choice of weaning strategy did not influence the development of the microbiomes post-weaning in either the rumen or feces of Holstein calves. This may result from the sequential development of the rumen to a more mature state with age (Li et al., 2012), and as all calves had access to solid feed prior to weaning, the fermentative role of the rumen in digestion had already started. Subsequent development may have resulted in the rumen harboring sufficient microbial diversity to perform all major fermentative and metabolic functions prior to weaning, and as such, the level of stress associated with the varying temporal nature of the dietary shift did not appear to have an effect on the microbial communities of weaned calves, as shown by the similar diversity and microbial compositions displayed in rumen and fecal microbiomes of gradually or abruptly weaned calves.

Weaning, however, affected the microbial diversity of the GI tract. OTU-level richness indices ACE and Chao1 were different between pre- and post-weaned calves, as were measures of evenness, such as the Shannon and Simpson indices (**Table 1**). Ruminal microbiota of weaned calves became less diverse and more uneven as a result of weaning, whereas fecal microbiota had a greater richness and evenness post-weaning. A parallel study to this work (Steele et al., 2015) showed that across treatments starter intake increased from 175 g/d pre-weaning to 1,648 g/d post-weaning. Similarly, the straw intake increased from 11.5 g/d pre-weaning to 75 g/d post-weaning. This significant dietary shift boosted the fermentation activities in the rumen resulting in increased ruminal concentration of VFA from 40 mM pre-weaning to more than 75 mM post-weaning and reduced rumen pH from 6.25 pre-weaning to less than 5.8 post-weaning. The introduction of solid feed post-weaning was expected to promote rumen bacterial diversity (Krause and Khafipour, 2010). However, a contrasting pattern was observed in this study, perhaps due to extensive fermentation activities and a low, fluctuating rumen pH during the post-weaning period (Steele et al., 2015). This environment was perhaps less favorable for some members of rumen microbiota, and as such, microbial diversity declined. On the other hand, exposure to such a harsh environment over a longer period of time may facilitate the establishment of new or existing members of rumen microbiota that are capable of efficiently degrading a specific diet; in this case, high starch concentrate, and tolerating lower and fluctuating rumen pH. As such, the diversity of the rumen microbiota may increase over time when microbiota becomes more mature and stable as the animal is aging (Jami et al., 2013). Our data are supported by previous studies showing that addition of starch to the diet reduced ruminal bacterial diversity in Holstein cows (Zened et al., 2013).

In contrast to rumen microbiota, fecal microbiota had a greater richness and evenness post-weaning. We speculate that higher intake of solid feed post-weaning increased the amount of substrates that passed to the lower intestines. As the hindgut is more stable and its pH is less variable, the greater availability of substrate could promote bacterial diversity (Khafipour et al., 2011).

In agreement with previous studies in cattle (Jami et al., 2013) and humans (Koropatkin et al., 2012), our results suggest that the developing gut (pre-weaning) comprises the same dominant phyla (Bacteroidetes, Firmicutes, and Proteobacteria) as the more mature gut, although the relative abundances vary with stage of development. In our study, Bacteroidetes, Firmicutes and Proteobacteria accounted for a total of 95.3 and 96.9% in the rumen and 95.3 and 92.2% in the feces of pre- and post-weaned calves, respectively. In the rumen, the p. Bacteroidetes, although still the dominant phylum, was less abundant in weaned calves. Bacteroidetes was also the dominant phylum in pre-weaned calves at 2 months of age that were fed milk replacer with access to calf starter, and weaned calves in a study by Jami et al. (2013), and although the abundances were stable between the two different group of animals, composition of the phyla were more reflective of those of the post- and pre-weaned calves in the current study, respectively. Members of p. Bacteroidetes have higher mean glycoside hydrolases (GHs) and polysaccharide lyases (PLs) genes per genome, as well as signal peptide-containing GHs and PLs, compared to the members of the p. Firmicutes, or any other bacterial phyla in the GI tract (El Kaoutari et al., 2013). As such, members of p. Bacteroidetes are one of the primary degraders of the many complex polysaccharides in the plant cell wall due to a greater range of GHs and PLs that are not present or poorly represented in members of other gut bacterial phyla (Henderson et al., 2015). During post-weaning when calves are fed higher amounts of starchy grains, the competitiveness of the members of p. Bacteroidetes in the microbial community declines allowing Firmicutes and other opportunistic phyla, such as p. Proteobacteria, to proliferate faster per unit of time resulting in an increase in the proportions of Firmicutes and Proteobacteria (Russell, 2007; Khafipour et al., 2009). Our results correspond to a previous study by Rey et al. (2013) who found Bacteroidetes was the most abundant phyla from days 19 to 83 in pre-weaned calves and showed no changes in its abundance, despite increasing consumption of starter and hay during this period, suggesting that consumption of starter increases the abundance of Bacteroidetes, but the level of starter intake does not alter its abundance within the rumen. The shifting carbohydrate composition of the diet during the transition from milk replacer to high starch starter is the key in changing the ratio of Bacteroidetes to Firmicutes in the rumen.

In the rumen, p. Bacteroidetes was dominated by the g. *Prevotella* regardless of developmental stage, whereas in the feces, g. *Bacteroides* was prominent, despite decreasing in abundance in post-weaned calves (Supplementary Tables 1 and 3). The dominance of g. *Prevotella* in the mature ruminal community has been reported previously in cattle (Stevenson and Weimer, 2007; Jami and Mizrahi, 2012; Jami et al., 2013) and in the human distal gut and in fecal microbiota (Gill et al., 2006). Similarly, the recent survey of the rumen and foregut microbial communities in 742 samples collected from 32 ruminant species across 35 countries from different geographical regions (Henderson et al., 2015) reported that most abundant and prevalent taxa within the rumen microbiota was g. *Prevotella*. As the rumen microbiome shifts toward that of a mature state and our calves had begun to consume starter prior to weaning, it was not unexpected that g. *Prevotella* was present in the pre-weaned rumen. The g. *Bacteroides* was also present in the rumen of calves at both developmental stages, decreasing post-weaning, similar to that observed by Wu et al. (2012). This also corresponds to a study by De Filippo et al. (2010), which explored the gut microbiota of children from Europe and rural Africa. In European children consuming a high caloric diet of animal protein, sugar, starch and fat, p. Bacteroidetes was dominated by g. *Bacteroides*, whereas g. *Prevotella* was the dominant member of this phylum in the gut of African children that consumed a diet of mainly plant fiber. The shifting bacterial composition of the rumen in the current study indicates that g. *Bacteroides* was present at a greater number due to the consumption of high caloric milk replacer in un-weaned calves, but decreased in abundances once calves were exposed to a higher starch diet post-weaning. Similarly, the relative abundance of g. *Bacteroides* decreased and an increase in g. *Prevotella* occurred in the fecal microbiota as the source of nutrients shifted away from milk replacer toward a high fiber diet. The results of these studies indicate that high fiber diets are conducive to the proliferation of specific fiber-degrading taxa.

In cattle, p. Fibrobacteres, which includes two species, *Fibrobacter succinogenes* and *Fibrobacter intestinalis*, is one of the main cellulolytic phyla in the rumen and hindgut (Jami et al., 2013; Henderson et al., 2015). In our study, however, this phylum was found at a very low abundance in post-weaned calves (0.53%) and was similar to pre-weaning abundances (0.22%; **Figure 2**). The abundance of this phylum is highly variable among animals and diets and has been shown to be completely absent from the fiber-adherent microbiome (Singh et al., 2014) and pooled rumen liquid microbiome of three Angus Simmental cross steers (Brulc et al., 2009). Even in cows fed a diet of 50% fiber, it was only present at 0.48% of the community (Jami and Mizrahi, 2012). In the recent global survey by Henderson et al. (2015), g. *Fibrobacter* was shown to be abundant in forage-fed cattle and its abundance declined in concentrate-fed animals indicating that perhaps that members of this phyla are only required to be present at a low relative abundance to play their key functional role in the GI tract (Henderson et al., 2015).

In a previous study (Rey et al., 2013), the presence of the g. *Succinivibrio* in the rumen of 3–12 days-old calves was positively correlated with concentrate intake and VFA concentration. Here, we observed no correlation with physiological variables (e.g., concentrations of individual and total VFA) in the rumen, but observed a positive correlation with BW, and starter and forage intake with fecal *Succinivibrio*. Additionally, several species of *Succinivibrio* have been studied in ruminants (Hernandez-Sanabria et al., 2012; Santos and Thompson, 2014) with *Succinivibrio dextrinosolvens* considered to predominate when feeding high-starch diets (O’Herrin and Kenealy, 1993). As the major products of *S. dextrinosolvens* include acetate and succinate (a precursor of propionate; Russell and Hespell, 1981), the correlation observed between *Succinivibrio* and the production of acetate, propionate and total VFA suggests *Succinivibrio* sp. may be involved in propionate production in ruminants (Hernandez-Sanabria et al., 2012).

Present in the rumen shortly after birth, the g. *Ruminococcus* can be found at increasing abundance through to maturity (Jami et al., 2013). In the current study, the relative abundance of g. *Ruminococcus* increased in the feces of weaned calves (**Table 3**) and showed a strong positive correlation with calf BW and intake of starter and forage (**Figure 4B**). This likely reflects the cellulolytic capabilities of members of this genus, such as *R. flavefaciens* and *R. albus* which are commonly found in the adult rumen (Flint et al., 2008; Jami et al., 2013).

Based on the predicted metagenomes (PICRUSt) of rumen and feces microbiomes, we inferred that there were several functional pathways associated with weaning. The significant reduction in genes associated with amino acid and carbohydrate metabolism in the feces microbiome in weaned calves reflects the shift in nutrient metabolism away from the lower GI tract to ruminal fermentation. However, increases in the rumen were not of equal size, accounting for ∼10.75% of all OTUs assigned to a KO, and did not show significant changes in carbohydrate metabolism (*P* > 0.05), perhaps indicating that the functional maturity of the rumen precedes microbial maturity. This corresponds with Li et al. (2012) who noted that carbohydrate transport and metabolism as indicated by the Clusters of Orthologous Groups (COGs) classification did not change with age, but was in fact present in ∼7.5% of COG assignments in pre-weaned calves, despite the absence of starter. The consumption of starter prior to weaning in the current study resulted in the proliferation of microbial taxa that would reside in the mature rumen, lessening the severity of the shift at weaning. That being said, it is important to note that PICRUSt predictions are based on known functions of the microbial communities present in the GI tract of humans and animals obtained from the whole genome shotgun sequencing of such samples. As there are limited numbers of shotgun sequencing studies in ruminants, the functionality of the members of ruminant GI microbiota may be over- or underestimated in PICRUSt.

Overall, our results suggest that the rumen and intestinal microbiomes of calves shift toward the mature ruminant state, hastened by the introduction of the solid feeds, as they trigger ruminal fermentation. The consumption of solid feeds prior to weaning could thus, lessen the severity of the shift in microbial community which occurs at weaning, as many taxa indicative of the mature state are already present. The predicted functional roles of these communities also appear to represent that of the mature gastrointestinal system even prior to weaning, suggesting a state of readiness and functional maturity. Additionally, we showed that weaning calves at 48 days of age using either a gradual or abrupt weaning strategy does not affect the development of their ruminal and fecal microbiomes and therefore, may not account for the declines in gain and intakes observed in calves during weaning.

## Author Contributions

SL, PA, HD, JP, EK, and MS made substantial contributions to the conception or design of the work. HD performed sequencing. SM and SL conducted data analysis and all authors were involved in the interpretation of data. SM and SL drafted the work and PA, HD, JP, EK, and MS revised it critically for important intellectual content. All authors approved the submitted versions and agree to be accountable for all aspects of the work ensuring that questions related to the accuracy or integrity of any part of the work were appropriately investigated and resolved.

## Conflict of Interest Statement

The authors declare that the research was conducted in the absence of any commercial or financial relationships that could be construed as a potential conflict of interest.

## Footnotes

1 http://www.ncbi.nlm.nih.gov/sra

2 https://cran.r-project.org/web/packages/corrplot/index.html

## References

[B1] Anderson M. (2005). *PERMANOVA: a FORTRAN Computer Program for Permutational Multivariate Analysis of Variance.* Department of Statistics, University of Auckland Auckland.

[B2] Biesheuvel M. H. Bijker P. G. H. Urlings H. A. P. (1991). Some aspects of the gastrointestinal microflora of veal calves fed different rations - a pilot-study. *Vet. Q.* 13 97–104. 10.1080/01652176.1991.9694291 1909065

[B3] Black J. L. Sharkey M. J. (1970). Reticular groove (sulcis reticuli): an obligatory adaptation in ruminant-like herbivores. *Mammalia* 34 294–302. 10.1515/mamm.1970.34.2.294

[B4] Brulc J. M. Antonopoulos D. A. Berg Miller M. E. Wilson M. K. Yannarell A. C. Dinsdale E. A. (2009). Gene-centric metagenomics of the fiber-adherent bovine rumen microbiome reveals forage specific glycoside hydrolases. *Proc. Natl. Acad. Sci. U.S.A.* 106 1948–1953. 10.1073/pnas.0806191105 19181843 PMC2633212

[B5] Bryant M. P. Small N. Bouma C. Robinsona I. (1958). Studies on the composition of the ruminal flora and fauna of young calves. *J. Dairy Sci.* 41 1747–1767. 10.3168/jds.S0022-0302(58)91160-3

[B6] Canadian Council on Animal Care (1993). *Guide to the Care and use of Experimental Animals* Vol. 1 2nd Edn eds Olfert E. D. Cross B. M. Mcwilliam A. A. (Ottawa, ON: Canadian Council on Animal Care).

[B7] Caporaso J. G. Bittinger K. Bushman F. D. Desantis T. Z. Andersen G. L. Knight R. (2010a). PyNAST: a flexible tool for aligning sequences to a template alignment. *Bioinformatics* 26 266–267. 10.1093/bioinformatics/btp636 19914921 PMC2804299

[B8] Caporaso J. G. Kuczynski J. Stombaugh J. Bittinger K. Bushman F. D. Costello E. K. (2010b). QIIME allows analysis of high-throughput community sequencing data. *Nat. Methods* 7 335–336. 10.1038/nmeth.f.303 20383131 PMC3156573

[B9] Caporaso J. G. Lauber C. L. Walters W. A. Berg-Lyons D. Huntley J. Fierer N. (2012). Ultra-high-throughput microbial community analysis on the Illumina HiSeq and MiSeq platforms. *ISME J.* 6 1621–1624. 10.1038/ismej.2012.8 22402401 PMC3400413

[B10] Carcer D. A. Denman S. E. Mcsweeney C. Morrison M. (2011). Evaluation of subsampling-based normalization strategies for tagged high-throughput sequencing data sets from gut microbiomes. *Appl. Environ. Microbiol.* 77 8795–8798. 10.1128/AEM.05491-11 21984239 PMC3233110

[B11] Church D. C. (1976). *Digestive Physiology and Nutrition of Ruminants* 2nd Edn Vol. I Corvallis, OR: O&B Books.

[B12] De Filippo C. Cavalieri D. Di Paola M. Ramazzotti M. Poullet J. B. Massart S. (2010). Impact of diet in shaping gut microbiota revealed by a comparative study in children from Europe and rural Africa. *Proc. Natl. Acad. Sci. U.S.A.* 107 14691–14696. 10.1073/pnas.1005963107 20679230 PMC2930426

[B13] Derakhshani H. Tun H. M. Khafipour E. (2016). An extended single-index multiplexed 16S rRNA sequencing for microbial community analysis on MiSeq Illumina platforms. *J. Basic Microbiol.* 56 321–326. 10.1002/jobm.201500420 26426811

[B14] Drackley J. K. Pollard B. C. Dann H. M. Stamey J. A. (2007). First-lactation milk production for cows fed control or intensified milk replacer programs as calves. *J. Dairy Sci.* 90(Suppl. 1) 779.17235155

[B15] Eckburg P. B. Bik E. M. Bernstein C. N. Purdom E. Dethlefsen L. Sargent M. (2005). Diversity of the Human intestinal microbial flora. *Science* 308 1635–1638. 10.1126/science.1110591 15831718 PMC1395357

[B16] Eckert E. C. Brown E. Leslie K. E. DeVries T. J. Steele M. A. (2015). Weaning age impacts growth, feed intake and behavioral indicators of stress in Holstein calves fed an elevated plane of nutrition. *J. Dairy Sci.* 98 8315–8328. 10.3168/jds.2014-9062 26142851

[B17] Edgar R. C. Haas B. J. Clemente J. C. Quince C. Knight R. (2011). UCHIME improves sensitivity and speed of chimera detection. *Bioinformatics* 27 2194–2200. 10.1093/bioinformatics/btr381 21700674 PMC3150044

[B18] El Kaoutari A. Armougom F. Leroy Q. Vialettes B. Million M. Raoult D. (2013). Development and validation of a microarray for the investigation of the cazymes encoded by the human gut microbiome. *PLoS ONE* 8:e84033 10.1371/journal.pone.0084033 PMC3877134 24391873

[B19] Flint H. J. Bayer E. A. Rincon M. T. Lamed R. White B. A. (2008). Polysaccharide utilization by gut bacteria: potential for new insights from genomic analysis. *Nat. Rev. Micro.* 6 121–131. 10.1038/nrmicro1817 18180751

[B20] Fonty G. Gouet P. Jouany J. P. Senaud J. (1987). Establishment of the microflora and anaerobic fungi in the rumen of lambs. *J. Gen. Microbiol.* 133 1835–1843.

[B21] Geishauser T. (1993). An instrument for the collection and transfer of ruminal fluid and for the administration of water soluble drugs in adult cattle. *Bovine Practit.* 27 38–42.

[B22] Gill S. R. Pop M. Deboy R. T. Eckburg P. B. Turnbaugh P. J. Samuel B. S. (2006). Metagenomic analysis of the human distal gut microbiome. *Science* 312 1355–1359. 10.1126/science.1124234 16741115 PMC3027896

[B23] Hammer O. Harper D. Ryan P. (2012). Past: paleontological statistics software package for education and data analysis. *Paleont. Electron* 4:9.

[B24] Henderson G. Cox F. Ganesh S. (2015). Rumen microbial community composition varies with diet and host, but a core microbiome is found across a wide geographical range. *Sci. Rep.* 5:14567 10.1038/srep14567 PMC4598811 26449758

[B25] Hernandez-Sanabria E. Goonewardene L. A. Wang Z. Durunna O. N. Moore S. S. Guan L. L. (2012). Impact of feed efficiency and diet on adaptive variations in the bacterial community in the rumen fluid of cattle. *Appl. Environ. Microbiol.* 78 1203–1214. 10.1128/AEM.05114-11 22156428 PMC3273029

[B26] Hungate R. E. (1966). *The Rumen and Its Microbes.* New York, NY: Academic Press.

[B27] Jami E. Israel A. Kotser A. Mizrahi I. (2013). Exploring the bovine rumen bacterial community from birth to adulthood. *ISME J.* 7 1069–1079. 10.1038/ismej.2013.2 23426008 PMC3660679

[B28] Jami E. Mizrahi I. (2012). Composition and similarity of bovine rumen microbiota across individual animals. *PLoS ONE* 7:e33306 10.1371/journal.pone.0033306 PMC3303817 22432013

[B29] Kanehisa M. Goto S. (2000). KEGG: kyoto encyclopedia of genes and genomes. *Nucleic Acids Res.* 28 27–30. 10.1093/nar/28.1.27 10592173 PMC102409

[B30] Khafipour E. Li S. Plaizier J. C. Dowd S. E. Krause D. O. (2011). Microbiome analysis of the rumen, cecum, and feces of dairy cows with subacute ruminal acidosis. *J. Anim. Sci.* 89:489.

[B31] Khafipour E. Li S. Plaizier J. C. Krause D. O. (2009). Rumen microbiome composition determined using two nutritional models of subacute ruminal acidosis. *Appl. Environ. Microbiol.* 75 7115–7124. 10.1128/AEM.00739-09 19783747 PMC2786511

[B32] Khan M. A. Lee H. J. Lee W. S. Kim H. S. Kim S. B. Ki K. S. (2007). Pre- and postweaning performance of holstein female calves fed milk through step-down and conventional methods. *J. Dairy Sci.* 90 876–885. 10.3168/jds.S0022-0302(07)71571-0 17235164

[B33] Khan M. A. Weary D. M. Von Keyserlingk M. A. G. (2011). Invited review: effects of milk ration on solid feed intake, weaning, and performance in dairy heifers. *J. Dairy Sci.* 94 1071–1081. 10.3168/jds.2010-3733 21338773

[B34] Koropatkin N. M. Cameron E. A. Martens E. C. (2012). How glycan metabolism shapes the human gut microbiota. *Nat. Rev. Micro.* 10 323–335. 10.1038/nrmicro2746 PMC4005082 22491358

[B35] Krause D. O. Khafipour E. (2010). “The fecal environment, the gut,” in *The Fecal Bacteria* eds Sadowski M. J. RL Whitman R. L. (Washington, DC: ASM Press) 1–21.

[B36] Kuczynski J. Costello E. K. Nemergut D. R. Zaneveld J. Lauber C. L. Knights D. (2010). Direct sequencing of the human microbiome readily reveals community differences. *Genome Biol.* 11:210 10.1186/gb-2010-11-5-210 PMC2898070 20441597

[B37] Langille M. G. I. Zaneveld J. Caporaso J. G. Mcdonald D. Knights D. Reyes J. A. (2013). Predictive functional profiling of microbial communities using 16S rRNA marker gene sequences. *Nat. Biotechnol.* 31 814–821. 10.1038/nbt.2676 23975157 PMC3819121

[B38] Li R. W. Connor E. E. Li C. Baldwin V. I. R. L. Sparks M. E. (2012). Characterization of the rumen microbiota of pre-ruminant calves using metagenomic tools. *Environ. Microbiol.* 14 129–139. 10.1111/j.1462-2920.2011.02543.x 21906219

[B39] Love M. I. Huber W. Anders S. (2014). Moderated estimation of fold change and dispersion for RNA-seq data with DESeq2. *Genome Biol.* 15:550 10.1186/s13059-014-0550-8 PMC4302049 25516281

[B40] Lozupone C. Knight R. (2005). UniFrac: a new phylogenetic method for comparing microbial communities. *Appl. Environ. Microbiol.* 71 8228–8235. 10.1128/AEM.71.12.8228-8235.2005 16332807 PMC1317376

[B41] Lozupone C. Lladser M. E. Knights D. Stombaugh J. Knight R. (2011). UniFrac: an effective distance metric for microbial community comparison. *ISME J.* 5 169–172. 10.1038/ismej.2010.133 20827291 PMC3105689

[B42] Masella A. P. Bartram A. K. Truszkowski J. M. Brown D. G. Neufeld J. D. (2012). PANDAseq: paired-end assembler for illumina sequences. *BMC Bioinformat.* 13:31 10.1186/1471-2105-13-31 PMC3471323 22333067

[B43] McDonald D. Price M. N. Goodrich J. Nawrocki E. P. Desantis T. Z. Probst A. (2012). An improved Greengenes taxonomy with explicit ranks for ecological and evolutionary analyses of bacteria and archaea. *ISME J.* 6 610–618. 10.1038/ismej.2011.139 22134646 PMC3280142

[B44] McMurdie P. J. Holmes S. (2013). phyloseq: an R package for reproducible interactive analysis and graphics of microbiome census data. *PLoS ONE* 8:e61217 10.1371/journal.pone.0061217 PMC3632530 23630581

[B45] Minato H. Otsuka M. Shirasaka S. Itabashi H. Mitsumori M. (1992). Colonization of microorganisms in the rumen of young calves. *J. Gen. Appl. Microbiol.* 38 447–456. 10.2323/Jgam.38.447

[B46] O’Herrin S. M. Kenealy W. R. (1993). Glucose and carbon dioxide metabolism by Succinivibrio dextrinosolvens. *Appl. Environ. Microbiol.* 59 748–755. 8481001 10.1128/aem.59.3.748-755.1993 PMC202185

[B47] Price M. N. Dehal P. S. Arkin A. P. (2009). FastTree: computing large minimum evolution trees with profiles instead of a distance matrix. *Mol. Biol. Evol.* 26 1641–1650. 10.1093/molbev/msp077 19377059 PMC2693737

[B48] Rey M. Enjalbert F. Combes S. Cauquil L. Bouchez O. Monteils V. (2013). Establishment of ruminal bacterial community in dairy calves from birth to weaning is sequential. *J. Appl. Microbiol.* 116 245–257. 10.1111/jam.12405 24279326

[B49] Roesch L. F. Fulthorpe R. R. Riva A. Casella G. Hadwin A. K. Kent A. D. (2007). Pyrosequencing enumerates and contrasts soil microbial diversity. *ISME J.* 1 283–290. 10.1038/ismej.2007.53 18043639 PMC2970868

[B50] Russell J. B. (2007). The energy spilling reactions of bacteria and other organisms. *J. Mol. Microbiol. Biotechnol.* 13 1–11. 10.1159/000103591 17693707

[B51] Russell J. B. Hespell R. B. (1981). Microbial rumen fermentation. *J. Dairy Sci.* 64 1153–1169. 10.3168/jds.S0022-0302(81)82694-X 7024344

[B52] Santiago A. Panda S. Mengels G. Martinez X. Azpiroz F. Dore J. (2014). Processing faecal samples: a step forward for standards in microbial community analysis. *BMC Microbiol.* 14:112 10.1186/1471-2180-14-112 PMC4021188 24884524

[B53] Santos E. Thompson F. (2014). “The Family Succinivibrionaceae,” in *The Prokaryotes* eds Rosenberg E. Delong E. Lory S. Stackebrandt E. Thompson F. (Berlin: Springer) 639–648.

[B54] Singh K. M. Reddy B. Patel D. Patel A. K. Parmar N. Patel A. (2014). High potential source for biomass degradation enzyme discovery and environmental aspects revealed through metagenomics of Indian buffalo Rumen. *Biol. Med. Res. Int.* 2014:267189 10.1155/2014/267189 PMC4124647 25136572

[B55] Soberon F. Van Amburgh M. E. (2013). LACTATION BIOLOGY SYMPOSIUM: The effect of nutrient intake from milk or milk replacer of preweaned dairy calves on lactation milk yield as adults: a meta-analysis of current data. *J. Anim. Sci.* 91 706–712. 10.2527/jas.2012-5834 23296823

[B56] Steele M. A. Leal L. E. Soberon F. Doelman J. H. Carson M. Metcalf J. A. (2015). Gradual weaning affects pre and post-weaning feed intake, gorwth and gastrointestinal develpoment in Holstein calves fed and elevated plane of nutrition during the pre-wenaing period. *J. Anim. Sci.* 98:242 10.3168/jds.2014-9062

[B57] Stevenson D. M. Weimer P. J. (2007). Dominance of Prevotella and low abundance of classical ruminal bacterial species in the bovine rumen revealed by relative quantification real-time PCR. *Appl. Microbiol. Biotechnol.* 75 165–174. 10.1007/s00253-006-0802-y 17235560

[B58] Sweeney B. C. Rushen J. Weary D. M. Passillé A. M. D. (2010). Duration of weaning, starter intake, and weight gain of dairy calves fed large amounts of milk. *J. Dairy Sci.* 93 148–152. 10.3168/jds.2009-2427 20059913

[B59] Wang Q. Garrity G. M. Tiedje J. M. Cole J. R. (2007). Naive Bayesian classifier for rapid assignment of rRNA sequences into the new bacterial taxonomy. *Appl. Environ. Microbiol.* 73 5261–5267. 10.1128/AEM.00062-07 17586664 PMC1950982

[B60] Warwick R. Clarke K. (2006). *PRIMER 6.* PRIMER-E Ltd Plymouth.

[B61] Wu S. Baldwin R. L. Li W. Li C. Connor E. E. Li R. W. (2012). The bacterial community composition of the bovine rumen detected using pyrosequencing of 16S rRNA Genes. *Metagenomics* 1 1–11. 10.4303/mg/235571

[B62] Zened A. Combes S. Cauquil L. Mariette J. Klopp C. Bouchez O. (2013). Microbial ecology of the rumen evaluated by 454 GS FLX pyrosequencing is affected by starch and oil supplementation of diets. *FEMS Microbiol. Ecol.* 83 504–514. 10.1111/1574-6941.12011 22974422

