# Supplementary material for: Development of Ruminal and Fecal Microbiomes Are Affected by Weaning But Not Weaning Strategy in Dairy Calves
Source: Front Microbiol. 2016 May 3;7:582. doi: 10.3389/fmicb.2016.00582 (PMC4853645; doi:10.3389/fmicb.2016.00582)
Supplement: Supplementary file 1 [file Table_1.DOCX]

Supplementary Material

**Development of ruminal and fecal microbiomes are not affected by weaning strategy in dairy calves**

**S.J. Meale^1†‡^, S.C. Li^2†^, P. Azavedo^2^, H. Derakhshani^2^, J.C. Plaizier^2^, E. Khafipour^2,3,*^ and M.A. Steele^1,*^**

*** Co-corresponding authors:**

**M.A. Steele**, Department of Agricultural, Food and Nutritional Science, University of Alberta, Edmonton, AB, Canada.

**E. Khafipour**, Department of Animal Science; University of Manitoba, Winnipeg, MB, R3T 2N2, Canada; Tel: 204-474-6112; Fax: 204-474-7628; Email: Ehsan.Khafipour@umanitoba.ca

# Supplementary Tables

| **Table S1.** Mean abundances of all ruminal taxa which were not different (P>0.05) between pre- *vs.* post-weaned calves | | | | | | |
| --- | --- | --- | --- | --- | --- | --- |
| **Phylum** | **Taxa^1^** | **Pre- weaning** | **Post-weaning** | **log2 FC** | **SE^2^** | **P-value^3^** |
| Acidobacteria | f. Acidobacteriaceae | 0.0001 | 0.0000 | -0.08 | 3.048 | NA |
|  | c.Chloracidobacteria | 0.0000 | 0.0003 | 0.10 | 3.044 | NA |
|  | o.Acidimicrobiales | 0.0001 | 0.0000 | -0.09 | 3.048 | NA |
|  | g. *Actinobaculum* | 0.0001 | 0.0000 | -0.09 | 3.048 | NA |
|  | g. *Actinomyces* | 0.0074 | 0.0001 | -0.29 | 3.033 | 0.975 |
|  | g. *Arcanobacterium* | 0.0002 | 0.0002 | -0.09 | 3.041 | NA |
|  | f. Actinomycetaceae | 0.0015 | 0.0000 | -0.41 | 3.038 | NA |
|  | g. *Parascardovia* | 0.0000 | 0.0001 | 0.07 | 3.049 | NA |
|  | g. *Brevibacterium* | 0.0002 | 0.0001 | -0.04 | 3.045 | NA |
|  | g. *Pseudoclavibacter* | 0.0000 | 0.0003 | 0.19 | 3.045 | NA |
|  | g. *Corynebacterium* | 0.0071 | 0.0033 | -1.32 | 0.681 | 0.086 |
|  | g. *Brachybacterium* | 0.0001 | 0.0001 | -0.04 | 3.045 | NA |
|  | f. Dermatophilaceae | 0.0001 | 0.0000 | -0.10 | 3.048 | NA |
|  | g. *Dietzia* | 0.0035 | 0.0007 | -0.46 | 2.996 | NA |
|  | g. *Leucobacter* | 0.0010 | 0.0001 | -0.38 | 3.029 | NA |
|  | f. Microbacteriaceae | 0.0002 | 0.0000 | -0.18 | 3.045 | NA |
|  | g. *Arthrobacter* | 0.0009 | 0.0002 | -0.64 | 3.024 | NA |
|  | g. *Kocuria* | 0.0018 | 0.0015 | -0.62 | 2.296 | NA |
|  | g. *Microbispora* | 0.0001 | 0.0000 | -0.11 | 3.049 | NA |
|  | f. Micrococcaceae | 0.0003 | 0.0000 | -0.26 | 3.041 | NA |
|  | g. *Rothia* | 0.0000 | 0.0002 | 0.08 | 3.048 | NA |
|  | o. Actinomycetales | 0.0029 | 0.0004 | -1.04 | 2.994 | NA |
|  | g. *Rhodococcus* | 0.0006 | 0.0004 | -0.28 | 3.021 | NA |
|  | g. *Sanguibacter* | 0.0000 | 0.0001 | 0.06 | 3.048 | NA |
|  | f. Bifidobacteriaceae | 0.0001 | 0.0157 | 0.46 | 3.033 | 0.938 |
|  | o. Bifidobacteriales | 0.0003 | 0.0002 | -0.05 | 3.038 | NA |
|  | c. Actinobacteria | 0.0001 | 0.0000 | -0.10 | 3.049 | NA |
| Armatimonadetes | g. *Fimbriimonas* | 0.0001 | 0.0000 | -0.11 | 3.049 | NA |
| Bacteroidetes | f. [Odoribacteraceae] | 0.0004 | 0.0000 | -0.27 | 3.041 | NA |
|  | g. *YRC22* | 0.2716 | 0.2490 | -0.68 | 0.439 | 0.174 |
|  | g. *5-7N15* | 0.0003 | 0.0000 | -0.11 | 3.048 | NA |
|  | f. Porphyromonadaceae | 0.0011 | 0.0002 | -0.64 | 3.024 | NA |
|  | g. *Paludibacter* | 0.0192 | 0.0272 | 0.61 | 3.016 | 0.911 |
|  | g. *Porphyromonas* | 0.0794 | 0.0007 | -0.70 | 2.993 | 0.911 |
|  | g. *Prevotella* | 36.2893 | 32.6915 | -0.14 | 0.191 | 0.606 |
|  | f. S24-7 | 1.2181 | 0.5217 | 0.51 | 0.333 | 0.177 |
|  | c. Bacteroidia | 0.0003 | 0.0000 | -0.13 | 3.048 | NA |
|  | f. Cryomorphaceae | 0.0002 | 0.0003 | 0.04 | 3.041 | NA |
|  | g. *Chryseobacterium* | 0.0006 | 0.0019 | 0.53 | 3.010 | NA |
|  | g. *Flavobacterium* | 0.0034 | 0.0016 | -0.63 | 1.252 | 0.746 |
|  | g. *Myroides* | 0.0024 | 0.0000 | -0.85 | 3.020 | NA |
|  | f. Flavobacteriaceae | 0.0063 | 0.0031 | -1.04 | 0.681 | 0.181 |
|  | g. *Wautersiella* | 0.0055 | 0.0044 | -2.10 | 1.286 | 0.156 |
|  | f. Chitinophagaceae | 0.0006 | 0.0017 | 0.58 | 3.006 | NA |
|  | f. Flexibacteraceae | 0.0001 | 0.0000 | -0.09 | 3.048 | NA |
|  | o. Sphingobacteriales | 0.0000 | 0.0002 | 0.19 | 3.046 | NA |
|  | f. Sphingobacteriaceae | 0.0086 | 0.0006 | -1.93 | 2.982 | 0.652 |
|  | g. *Pedobacter* | 0.0008 | 0.0008 | -0.12 | 3.022 | NA |
| Chloroflexi | g. *SHD-231* | 0.0088 | 0.0004 | -0.66 | 3.020 | 0.911 |
| Cyanobacteria | c. 4C0d-2 | 0.0000 | 0.0002 | 0.07 | 3.048 | NA |
|  | o. YS2 | 0.0980 | 0.4672 | 0.94 | 0.443 | 0.057 |
|  | o. CAB-I | 0.0005 | 0.0000 | -0.11 | 3.048 | NA |
| Deferribacteres | g. *Mucispirillum* | 0.0066 | 0.0058 | -0.02 | 1.700 | 0.991 |
| Elusimicrobia | c. Endomicrobia | 0.0024 | 0.0001 | -0.46 | 3.031 | NA |
| Firmicutes | g. *Bacillus* | 0.0002 | 0.0000 | -0.08 | 3.048 | NA |
|  | f. Bacillaceae | 0.0001 | 0.0001 | -0.03 | 3.044 | NA |
|  | o. Bacillales | 0.0003 | 0.0000 | -0.20 | 3.044 | NA |
|  | f. Planococcaceae | 0.0006 | 0.0003 | -0.05 | 3.033 | NA |
|  | g. *Jeotgalicoccus* | 0.0001 | 0.0003 | 0.06 | 3.040 | NA |
|  | f. Staphylococcaceae | 0.0017 | 0.0002 | -0.33 | 3.033 | NA |
|  | g. *Staphylococcus* | 0.0010 | 0.0014 | -0.28 | 3.014 | NA |
|  | g. *Exiguobacterium* | 0.0001 | 0.0000 | -0.11 | 3.049 | NA |
|  | g. *Aerococcus* | 0.0010 | 0.0002 | -0.41 | 3.025 | NA |
|  | g. *Facklamia* | 0.0007 | 0.0002 | -0.36 | 3.029 | NA |
|  | g. *Carnobacterium* | 0.0003 | 0.0001 | -0.15 | 3.041 | NA |
|  | g. *Desemzia* | 0.0001 | 0.0001 | -0.01 | 3.046 | NA |
|  | g. *Trichococcus* | 0.0001 | 0.0000 | -0.09 | 3.048 | NA |
|  | f. Enterococcaceae | 0.0001 | 0.0000 | -0.11 | 3.049 | NA |
|  | g. *Lactobacillus* | 0.0225 | 0.0271 | 0.54 | 0.334 | 0.156 |
|  | g. *Leuconostoc* | 0.0001 | 0.0000 | -0.11 | 3.049 | NA |
|  | g. *Weissella* | 0.0002 | 0.0000 | -0.09 | 3.048 | NA |
|  | o. Lactobacillales | 0.0007 | 0.0000 | -0.24 | 3.040 | NA |
|  | g. *Lactococcus* | 0.0001 | 0.0000 | -0.11 | 3.049 | NA |
|  | c. Bacilli | 0.0001 | 0.0001 | -0.02 | 3.048 | NA |
|  | g. *Turicibacter* | 0.0009 | 0.0001 | -0.13 | 3.041 | NA |
|  | f. Catabacteriaceae | 0.0102 | 0.0008 | -0.95 | 3.017 | 0.865 |
|  | g. *Anaerococcus* | 0.0002 | 0.0000 | -0.20 | 3.045 | NA |
|  | g. *Helcococcus* | 0.0004 | 0.0002 | -0.15 | 3.035 | NA |
|  | g. *Mogibacterium* | 0.0003 | 0.0003 | -0.02 | 3.037 | NA |
|  | f. Clostridiaceae | 0.2547 | 0.2928 | 0.10 | 0.168 | 0.666 |
|  | g. *Peptoniphilus* | 0.0001 | 0.0002 | -0.02 | 3.045 | NA |
|  | g. *Sarcina* | 0.0008 | 0.0010 | 0.17 | 3.018 | NA |
|  | g. *Dehalobacterium* | 0.0017 | 0.0006 | -0.60 | 3.000 | NA |
|  | f. Dehalobacteriaceae | 0.0002 | 0.0001 | -0.21 | 3.039 | NA |
|  | f. EtOH8 | 0.0010 | 0.0001 | -0.48 | 3.031 | NA |
|  | g. *Anaerofustis* | 0.0000 | 0.0001 | 0.07 | 3.049 | NA |
|  | g. *Eubacterium* | 0.0023 | 0.0000 | -0.67 | 3.026 | NA |
|  | f. Eubacteriaceae | 0.0000 | 0.0002 | 0.14 | 3.045 | NA |
|  | g. *[Ruminococcus]* | 0.0644 | 0.0715 | 0.13 | 0.377 | 0.858 |
|  | g. *Anaerostipes* | 0.1163 | 0.4814 | -0.23 | 0.503 | 0.778 |
|  | g. *Blautia* | 0.0166 | 0.0121 | 0.29 | 0.470 | 0.666 |
|  | g. *Dorea* | 0.0005 | 0.0004 | -0.21 | 3.018 | NA |
|  | g. *Lachnobacterium* | 0.0074 | 0.1356 | 0.15 | 3.006 | 0.991 |
|  | g. *Lachnospira* | 0.0049 | 0.0085 | 1.10 | 0.792 | 0.223 |
|  | g. *Moryella* | 0.3163 | 0.3419 | 0.44 | 0.253 | 0.125 |
|  | g. *Oribacterium* | 0.0000 | 0.0001 | 0.12 | 3.046 | NA |
|  | g. *Roseburia* | 0.2763 | 0.1949 | -0.72 | 0.389 | 0.101 |
|  | f. Peptococcaceae | 0.0001 | 0.0000 | -0.11 | 3.049 | NA |
|  | g. *Peptococcus* | 0.0007 | 0.0013 | 0.22 | 3.011 | NA |
|  | g. *rc4-4* | 0.0000 | 0.0001 | 0.07 | 3.049 | NA |
|  | f. Peptostreptococcaceae | 0.0283 | 0.0007 | -0.50 | 2.998 | 0.930 |
|  | g. *Anaerofilum* | 0.0002 | 0.0000 | -0.17 | 3.045 | NA |
|  | g. *Faecalibacterium* | 0.0296 | 0.0029 | 0.27 | 0.636 | 0.785 |
|  | g. *Anaerovibrio* | 0.0401 | 0.0504 | 0.01 | 0.430 | 0.991 |
|  | g. *Phascolarctobacterium* | 0.0691 | 0.0389 | -1.01 | 0.542 | 0.098 |
|  | g. *Selenomonas* | 0.0245 | 0.0151 | -0.70 | 0.482 | 0.205 |
|  | g. *Veillonella* | 0.0086 | 0.0003 | -0.60 | 3.014 | 0.911 |
|  | g. *Adlercreutzia* | 0.0026 | 0.0009 | -1.41 | 1.183 | NA |
|  | g. *Atopobium* | 0.0114 | 0.0057 | -0.05 | 1.608 | 0.991 |
|  | g. *Collinsella* | 0.0001 | 0.0003 | 0.05 | 3.041 | NA |
|  | g. *Eggerthella* | 0.0005 | 0.0001 | -0.28 | 3.034 | NA |
|  | f. Coriobacteriaceae | 2.3691 | 3.5253 | 0.42 | 0.260 | 0.156 |
|  | g. *Olsenella* | 0.0612 | 0.0712 | 0.25 | 0.281 | 0.498 |
|  | g. *Slackia* | 0.0001 | 0.0001 | -0.03 | 3.046 | NA |
|  | c. Clostridia | 0.4143 | 0.3191 | -0.26 | 0.153 | 0.141 |
|  | g. *Catenibacterium* | 0.0001 | 0.0005 | 0.25 | 3.038 | NA |
|  | g. *Coprobacillus* | 0.0000 | 0.0001 | 0.04 | 3.049 | NA |
|  | g. *Erysipelothrix* | 0.0001 | 0.0000 | -0.13 | 3.049 | NA |
|  | g. *Holdemania* | 0.0005 | 0.0002 | -0.19 | 3.037 | NA |
|  | f. Erysipelotrichaceae | 0.0050 | 0.0012 | -1.38 | 2.486 | 0.708 |
|  | o. Erysipelotrichales | 0.0007 | 0.0033 | 0.72 | 2.985 | NA |
| Fusobacteria | g. *Fusobacterium* | 0.0041 | 0.0014 | -1.87 | 1.173 | 0.163 |
|  | f. Fusobacteriaceae | 0.0010 | 0.0003 | -0.15 | 3.040 | NA |
|  | g. *Leptotrichia* | 0.0001 | 0.0001 | -0.03 | 3.045 | NA |
|  | f. Leptotrichiaceae | 0.0010 | 0.0001 | -0.50 | 3.027 | NA |
| Lentisphaerae | c. [Lentisphaeria] | 0.0003 | 0.0002 | -0.17 | 3.037 | NA |
|  | f. R4-45B | 0.0010 | 0.0000 | -0.50 | 3.034 | NA |
| Planctomycetes | f. Pirellulaceae | 0.0033 | 0.0000 | -0.85 | 3.022 | NA |
|  | g. *Planctomyces* | 0.0002 | 0.0000 | -0.12 | 3.048 | NA |
| Proteobacteria | o. BD7-3 | 0.0005 | 0.0000 | -0.21 | 3.044 | NA |
|  | g. *Brevundimonas* | 0.0034 | 0.0046 | -0.85 | 0.846 | 0.415 |
|  | g. *Mycoplana* | 0.0000 | 0.0001 | 0.05 | 3.048 | NA |
|  | f. Caulobacteraceae | 0.0011 | 0.0014 | 0.44 | 2.990 | NA |
|  | g. *Phenylobacterium* | 0.0001 | 0.0001 | 0.01 | 3.045 | NA |
|  | o. Ellin329 | 0.0002 | 0.0000 | -0.11 | 3.048 | NA |
|  | c. Alphaproteobacteria | 0.0028 | 0.0021 | -0.63 | 2.980 | 0.911 |
|  | o. RF32 | 0.3648 | 0.2245 | -0.79 | 0.515 | 0.177 |
|  | g. *Balneimonas* | 0.0000 | 0.0001 | 0.04 | 3.048 | NA |
|  | f. Brucellaceae | 0.0020 | 0.0000 | -1.44 | 3.011 | NA |
|  | g. *Ochrobactrum* | 0.0007 | 0.0001 | -0.25 | 3.034 | NA |
|  | g. *Devosia* | 0.0006 | 0.0006 | -0.25 | 3.023 | NA |
|  | g. *Parvibaculum* | 0.0001 | 0.0000 | -0.11 | 3.049 | NA |
|  | o. Rhizobiales | 0.0010 | 0.0003 | -0.46 | 3.014 | NA |
|  | f. Phyllobacteriaceae | 0.0022 | 0.0000 | -1.39 | 3.016 | NA |
|  | g. *Agrobacterium* | 0.0003 | 0.0004 | 0.10 | 3.033 | NA |
|  | f. Rhizobiaceae | 0.0003 | 0.0005 | 0.04 | 3.027 | NA |
|  | f. Rhodobacteraceae | 0.0011 | 0.0004 | -0.35 | 3.016 | NA |
|  | g. *Paracoccus* | 0.0023 | 0.0002 | -1.13 | 2.999 | NA |
|  | g. *Rhodobacter* | 0.0002 | 0.0001 | -0.09 | 3.041 | NA |
|  | g. *Roseomonas* | 0.0001 | 0.0000 | -0.13 | 3.049 | NA |
|  | g. *Azospirillum* | 0.0000 | 0.0002 | 0.10 | 3.049 | NA |
|  | f. mitochondria | 0.0003 | 0.0021 | 1.45 | 3.001 | NA |
|  | f. Erythrobacteraceae | 0.0002 | 0.0000 | -0.24 | 3.042 | NA |
|  | o. Sphingomonadales | 0.0006 | 0.0000 | -0.30 | 3.041 | NA |
|  | f. Sphingomonadaceae | 0.0003 | 0.0001 | -0.15 | 3.041 | NA |
|  | g. *Novosphingobium* | 0.0002 | 0.0001 | -0.12 | 3.041 | NA |
|  | g. *Sphingobium* | 0.0001 | 0.0004 | 0.04 | 3.041 | NA |
|  | g. *Sphingomonas* | 0.0005 | 0.0007 | 0.24 | 3.018 | NA |
|  | g. *Sphingopyxis* | 0.0007 | 0.0001 | -0.26 | 3.033 | NA |
|  | g. *Achromobacter* | 0.0001 | 0.0000 | -0.09 | 3.048 | NA |
|  | g. *Oligella* | 0.0015 | 0.0005 | -0.75 | 3.011 | NA |
|  | g. *Tetrathiobacter* | 0.0000 | 0.0001 | 0.07 | 3.049 | NA |
|  | g. *Lautropia* | 0.0021 | 0.0002 | -1.10 | 3.007 | NA |
|  | g. *Acidovorax* | 0.0014 | 0.0020 | 0.12 | 2.989 | NA |
|  | g. *Aquabacterium* | 0.0001 | 0.0015 | 0.24 | 3.035 | NA |
|  | g. *Comamonas* | 0.0029 | 0.0003 | -1.34 | 3.000 | NA |
|  | g. *Hylemonella* | 0.0005 | 0.0001 | -0.37 | 3.031 | NA |
|  | g. *Rubrivivax* | 0.0006 | 0.0015 | 0.51 | 3.009 | NA |
|  | o. Burkholderiales | 0.0025 | 0.0012 | -0.41 | 2.995 | NA |
|  | g. *Janthinobacterium* | 0.0006 | 0.0001 | -0.29 | 3.034 | NA |
|  | f. Oxalobacteraceae | 0.0004 | 0.0005 | -0.16 | 3.028 | NA |
|  | g. *Oxalobacter* | 0.0019 | 0.0010 | -0.31 | 3.010 | NA |
|  | o. MKC10 | 0.0012 | 0.0003 | -0.81 | 3.014 | NA |
|  | g. *Kingella* | 0.0000 | 0.0001 | 0.06 | 3.048 | NA |
|  | g. *Neisseria* | 0.0113 | 0.0033 | -1.09 | 0.542 | 0.071 |
|  | f. Bacteriovoracaceae | 0.0018 | 0.0007 | 0.15 | 3.029 | NA |
|  | g. *Bilophila* | 0.0032 | 0.0022 | -0.17 | 2.991 | 0.991 |
|  | f. Desulfovibrionaceae | 0.0051 | 0.0021 | -1.40 | 0.949 | 0.195 |
|  | o. Desulfovibrionales | 0.0006 | 0.0005 | -0.13 | 3.026 | NA |
|  | o. GMD14H09 | 0.0010 | 0.0003 | -0.18 | 3.026 | NA |
|  | f. Haliangiaceae | 0.0000 | 0.0002 | 0.05 | 3.048 | NA |
|  | c. Deltaproteobacteria | 0.0010 | 0.0002 | -0.46 | 3.028 | NA |
|  | g. *Arcobacter* | 0.0001 | 0.0000 | -0.13 | 3.049 | NA |
|  | f. Campylobacteraceae | 0.0001 | 0.0000 | -0.12 | 3.049 | NA |
|  | g. *Helicobacter* | 0.0092 | 0.0211 | 0.21 | 1.015 | 0.911 |
|  | f. Helicobacteraceae | 0.0001 | 0.0002 | -0.03 | 3.044 | NA |
|  | f. Aeromonadaceae | 0.0003 | 0.0004 | 0.04 | 3.037 | NA |
|  | o. Aeromonadales | 0.0003 | 0.0015 | 0.71 | 3.011 | NA |
|  | g. *Anaerobiospirillum* | 0.0001 | 0.0003 | 0.09 | 3.041 | NA |
|  | f. [Chromatiaceae] | 0.0012 | 0.0007 | -0.16 | 3.033 | NA |
|  | g. *Rheinheimera* | 0.0000 | 0.0001 | 0.07 | 3.049 | NA |
|  | g. *Cellvibrio* | 0.0002 | 0.0001 | -0.02 | 3.045 | NA |
|  | f. Alteromonadaceae | 0.0005 | 0.0001 | -0.31 | 3.038 | NA |
|  | f. Idiomarinaceae | 0.0005 | 0.0000 | -0.53 | 3.036 | NA |
|  | g. *Shewanella* | 0.0004 | 0.0009 | 0.40 | 3.016 | NA |
|  | g. *Citrobacter* | 0.0001 | 0.0000 | -0.09 | 3.048 | NA |
|  | g. *Erwinia* | 0.0011 | 0.0010 | 0.19 | 2.996 | NA |
|  | g. *Escherichia* | 0.0022 | 0.0014 | -0.58 | 1.385 | NA |
|  | f. Enterobacteriaceae | 0.0029 | 0.0011 | -0.98 | 1.784 | NA |
|  | g. *Providencia* | 0.0003 | 0.0000 | -0.18 | 3.045 | NA |
|  | g. *Trabulsiella* | 0.0002 | 0.0001 | -0.04 | 3.044 | NA |
|  | g. *Legionella* | 0.0000 | 0.0002 | 0.12 | 3.045 | NA |
|  | g. *Halomonas* | 0.0000 | 0.0001 | 0.07 | 3.049 | NA |
|  | g. *Aggregatibacter* | 0.0010 | 0.0007 | -0.08 | 3.018 | NA |
|  | g. *Bibersteinia* | 0.0012 | 0.0007 | -0.10 | 3.017 | NA |
|  | g. *Gallibacterium* | 0.0112 | 0.0015 | -1.83 | 0.898 | 0.069 |
|  | g. *Haemophilus* | 0.0003 | 0.0000 | -0.32 | 3.042 | NA |
|  | g. *Mannheimia* | 0.0037 | 0.0003 | -0.47 | 3.029 | NA |
|  | g. *Pasteurella* | 0.0025 | 0.0003 | -0.39 | 3.037 | NA |
|  | g. *Alkanindiges* | 0.0006 | 0.0002 | 0.03 | 3.028 | NA |
|  | g. *Enhydrobacter* | 0.0003 | 0.0001 | -0.26 | 3.033 | NA |
|  | g. *Moraxella* | 0.0004 | 0.0001 | -0.18 | 3.037 | NA |
|  | f. Moraxellaceae | 0.0015 | 0.0005 | -0.74 | 3.006 | NA |
|  | f. Pseudomonadaceae | 0.0112 | 0.0044 | -0.53 | 0.644 | 0.532 |
|  | g. *Pseudomonas* | 0.0012 | 0.0012 | 0.23 | 3.011 | NA |
|  | o. PYR10d3 | 0.0001 | 0.0000 | -0.09 | 3.048 | NA |
|  | g. *Methylophaga* | 0.0009 | 0.0001 | -0.35 | 3.033 | NA |
|  | g. *Ignatzschineria* | 0.0003 | 0.0001 | -0.11 | 3.041 | NA |
|  | g. *Luteimonas* | 0.0005 | 0.0000 | -0.43 | 3.039 | NA |
|  | f. Xanthomonadaceae | 0.0008 | 0.0011 | 0.07 | 3.004 | NA |
|  | g. *Pseudoxanthomonas* | 0.0001 | 0.0002 | 0.25 | 3.036 | NA |
|  | g. *Stenotrophomonas* | 0.0041 | 0.0023 | -0.74 | 0.915 | 0.538 |
|  | g. *Xanthomonas* | 0.0001 | 0.0002 | -0.04 | 3.045 | NA |
| Spirochaetes | g. *Brachyspira* | 0.0000 | 0.0001 | 0.09 | 3.050 | NA |
|  | o. PL-11B10 | 0.0005 | 0.0001 | -0.12 | 3.041 | NA |
|  | c. Spirochaetes | 0.0044 | 0.0019 | -0.50 | 3.015 | NA |
|  | o. Sphaerochaetales | 0.0037 | 0.0001 | -1.12 | 3.011 | NA |
|  | g. *Sphaerochaeta* | 0.0011 | 0.0001 | -0.76 | 3.022 | NA |
|  | f. Spirochaetaceae | 0.0021 | 0.0038 | 0.03 | 2.997 | 0.991 |
|  | g. *Treponema* | 0.8005 | 0.5763 | -0.19 | 0.432 | 0.785 |
| Synergistetes | g. *Pyramidobacter* | 0.0976 | 0.0407 | -0.50 | 0.367 | 0.233 |
|  | o. Synergistales | 0.0002 | 0.0000 | -0.09 | 3.048 | NA |
|  | f. Synergistaceae | 0.0175 | 0.0003 | -0.96 | 3.014 | 0.865 |
|  | g. *Synergistes* | 0.0001 | 0.0001 | -0.05 | 3.042 | NA |
| Tenericutes | g. *Acholeplasma* | 0.0001 | 0.0000 | -0.09 | 3.048 | NA |
|  | g. *Anaeroplasma* | 0.0386 | 0.0030 | -0.66 | 2.997 | 0.911 |
|  | f. Anaeroplasmataceae | 0.0022 | 0.0001 | -1.85 | 2.999 | NA |
|  | o. Anaeroplasmatales | 0.0307 | 0.0527 | 1.49 | 0.686 | 0.051 |
|  | g. *Mycoplasma* | 0.0009 | 0.0031 | 0.81 | 2.987 | NA |
|  | c. Mollicutes | 0.0014 | 0.0004 | -0.42 | 3.008 | NA |
|  | o. RF39 | 0.1869 | 0.3414 | 0.73 | 0.347 | 0.061 |
| Thermi | g. *Deinococcus* | 0.0000 | 0.0001 | 0.05 | 3.048 | NA |
|  | g. *Meiothermus* | 0.0000 | 0.0001 | 0.08 | 3.049 | NA |
| TM7 | f. Rs-045 | 0.0000 | 0.0007 | 0.14 | 3.044 | NA |
| Verrucomicrobia | f. R4-41B | 0.0022 | 0.0000 | -0.19 | 3.045 | 0.991 |
|  | o. WCHB1-41 | 0.0001 | 0.0000 | -0.11 | 3.049 | NA |
|  | g. *Akkermansia* | 0.0006 | 0.0000 | -0.29 | 3.041 | NA |
|  | g. *Prosthecobacter* | 0.0000 | 0.0001 | 0.05 | 3.048 | NA |
| ^1^ Sequences that could not be assigned to a genus are displayed using the highest taxonomic level (class, c.; order, o.; family, f.) they could be assigned to. | | | | | | |
| ^2^ FC, fold change; SE, standard error for the log2 fold change estimate. | | | | | | |
| ^3^ If within a row, all samples have zero counts, the p-value will be "NA" | | | | | | |
