# Supplementary material for: Development of Ruminal and Fecal Microbiomes Are Affected by Weaning But Not Weaning Strategy in Dairy Calves
Source: Front Microbiol. 2016 May 3;7:582. doi: 10.3389/fmicb.2016.00582 (PMC4853645; doi:10.3389/fmicb.2016.00582)
Supplement: Supplementary file 2 [file Table_2.DOCX]

Supplementary Material

**Development of ruminal and fecal microbiomes are not affected by weaning strategy in dairy calves**

**S.J. Meale^1†‡^, S.C. Li^2†^, P. Azavedo^2^, H. Derakhshani^2^, J.C. Plaizier^2^, E. Khafipour^2,3,*^ and M.A. Steele^1,*^**

*** Co-corresponding authors:**

**M.A. Steele**, Department of Agricultural, Food and Nutritional Science, University of Alberta, Edmonton, AB, Canada.

**E. Khafipour**, Department of Animal Science; University of Manitoba, Winnipeg, MB, R3T 2N2, Canada; Tel: 204-474-6112; Fax: 204-474-7628; Email: Ehsan.Khafipour@umanitoba.ca

# Supplementary Tables

| **Table S2.** Mean abundances of all fecal taxa which were not different (P>0.05) between pre- *vs.* post-weaned calves | | | | | | |
| --- | --- | --- | --- | --- | --- | --- |
| **Phylum** | **Taxa^1^** | **Pre- weaning** | **Post-weaning** | **log2 FC** | **SE^2^** | **P-value^3^** |
| Actinobacteria | o. Acidimicrobiales | 0.0001 | 0.0000 | -0.90 | 3.048 | NA |
|  | f. ACK-M1 | 0.0000 | 0.0001 | -0.73 | 3.048 | NA |
|  | g. *Actinomyces* | 0.0006 | 0.0002 | -1.11 | 3.038 | NA |
|  | g. *Arcanobacterium* | 0.0001 | 0.0000 | -0.89 | 3.048 | NA |
|  | f. Actinomycetaceae | 0.0000 | 0.0002 | -0.73 | 3.048 | NA |
|  | g. *Parascardovia* | 0.0003 | 0.0003 | -0.84 | 3.036 | NA |
|  | g. *Corynebacterium* | 0.0019 | 0.0020 | -0.80 | 1.895 | NA |
|  | g. *Dietzia* | 0.0004 | 0.0003 | -1.10 | 3.035 | NA |
|  | f. Geodermatophilaceae | 0.0000 | 0.0002 | -0.66 | 3.044 | NA |
|  | g. *Leucobacter* | 0.0003 | 0.0001 | -0.97 | 3.038 | NA |
|  | g. *Kocuria* | 0.0002 | 0.0000 | -0.82 | 3.048 | NA |
|  | o. Actinomycetales | 0.0002 | 0.0002 | -0.71 | 3.040 | NA |
|  | f. Nocardioidaceae | 0.0000 | 0.0002 | -0.75 | 3.048 | NA |
|  | f. Streptomycetaceae | 0.0000 | 0.0002 | -0.73 | 3.048 | NA |
|  | f. Bifidobacteriaceae | 0.0014 | 0.0006 | -1.13 | 3.012 | NA |
|  | o. Bifidobacteriales | 0.0007 | 0.0002 | -1.02 | 3.019 | NA |
|  | o. NA | 0.0000 | 0.0001 | -0.73 | 3.048 | NA |
| Armatimonadetes | g. *Fimbriimonas* | 0.0001 | 0.0000 | -0.85 | 3.048 | NA |
| Bacteroidetes | f. [Barnesiellaceae] | 0.2972 | 0.1453 | -0.75 | 0.346 | 0.066 |
|  | g. *Butyricimonas* | 0.3177 | 0.3878 | 0.59 | 0.367 | 0.182 |
|  | f. [Odoribacteraceae] | 0.0008 | 0.0007 | -0.50 | 3.019 | NA |
|  | g. *Odoribacter* | 1.1381 | 0.8354 | -0.34 | 0.223 | 0.209 |
|  | f. [Paraprevotellaceae] | 0.0133 | 0.0109 | -0.02 | 0.459 | 0.969 |
|  | g. *YRC22* | 0.0004 | 0.0000 | -0.93 | 3.048 | NA |
|  | g. 5-*7N15* | 0.0002 | 0.0000 | -0.86 | 3.048 | NA |
|  | f. Bacteroidaceae | 0.1326 | 0.1160 | -0.06 | 0.251 | 0.877 |
|  | f. BS11 | 0.0002 | 0.0001 | -0.75 | 3.045 | NA |
|  | f. Porphyromonadaceae | 0.0095 | 0.0134 | 0.37 | 0.253 | 0.225 |
|  | g. *Paludibacter* | 0.0000 | 0.0001 | -0.73 | 3.048 | NA |
|  | g. *Parabacteroides* | 2.7146 | 2.8356 | 0.10 | 0.163 | 0.660 |
|  | g. *Porphyromonas* | 0.0001 | 0.0000 | -0.89 | 3.048 | NA |
|  | f. Prevotellaceae | 0.0003 | 0.0005 | -0.64 | 3.024 | NA |
|  | f. Rikenellaceae | 0.4414 | 0.4501 | -0.11 | 0.266 | 0.774 |
|  | g. *Flavobacterium* | 0.0002 | 0.0002 | -0.72 | 3.036 | NA |
|  | f. Flavobacteriaceae | 0.0001 | 0.0000 | -0.90 | 3.048 | NA |
|  | f. Sphingobacteriaceae | 0.0000 | 0.0001 | -0.68 | 3.049 | NA |
| Chlamydiae | f. Chlamydiaceae | 0.0021 | 0.0003 | -0.81 | 3.044 | NA |
| Chlorobi | o. NA | 0.0000 | 0.0001 | -0.73 | 3.048 | NA |
| Chloroflexi | g. *SHD-231* | 0.0011 | 0.0005 | -1.16 | 3.026 | NA |
|  | o. mle1-48 | 0.0001 | 0.0000 | -0.86 | 3.048 | NA |
| Cyanobacteria | o. NA | 0.0002 | 0.0001 | -0.83 | 3.044 | NA |
|  | o. YS2 | 0.1576 | 0.3089 | 0.76 | 0.381 | 0.094 |
|  | o. CAB-I | 0.0000 | 0.0002 | -0.75 | 3.048 | NA |
|  | o. Streptophyta | 0.0002 | 0.0001 | -0.82 | 3.045 | NA |
|  | o. NA | 0.0001 | 0.0001 | -0.86 | 3.045 | NA |
| Deferribacteres | g. *Mucispirillum* | 0.0136 | 0.0064 | -1.63 | 0.863 | 0.115 |
| Elusimicrobia | f. Elusimicrobiaceae | 0.0261 | 0.0468 | 1.03 | 0.492 | 0.077 |
| Fibrobacteres | g. *Fibrobacter* | 0.0001 | 0.0000 | -0.90 | 3.049 | NA |
| Firmicutes | f. Bacillaceae | 0.0001 | 0.0002 | -0.83 | 3.044 | NA |
|  | o. Bacillales | 0.0000 | 0.0005 | -0.50 | 3.041 | NA |
|  | f. Planococcaceae | 0.0005 | 0.0072 | -0.46 | 3.024 | 0.921 |
|  | g. *Shimazuella* | 0.0002 | 0.0000 | -0.90 | 3.048 | NA |
|  | g. *Aerococcus* | 0.0001 | 0.0001 | -0.82 | 3.044 | NA |
|  | g. *Enterococcus* | 0.0011 | 0.0003 | -1.27 | 3.028 | NA |
|  | g. *Pediococcus* | 0.0012 | 0.0002 | -1.42 | 3.023 | NA |
|  | g. *Fructobacillus* | 0.0005 | 0.0000 | -0.89 | 3.048 | NA |
|  | g. *Leuconostoc* | 0.0033 | 0.0001 | -2.34 | 3.005 | NA |
|  | g. *Lactococcus* | 0.0032 | 0.0003 | -2.85 | 2.992 | 0.479 |
|  | o. NA | 0.0020 | 0.0000 | -1.80 | 3.017 | NA |
|  | g. *Turicibacter* | 0.0022 | 0.0003 | -1.19 | 3.022 | NA |
|  | f. Catabacteriaceae | 0.0004 | 0.0028 | -0.11 | 3.010 | NA |
|  | g. *Mogibacterium* | 0.0000 | 0.0006 | -0.26 | 3.032 | NA |
|  | g. *Sarcina* | 0.0009 | 0.0007 | -0.80 | 3.015 | NA |
|  | g. *Dehalobacterium* | 0.0006 | 0.0011 | -0.60 | 3.010 | NA |
|  | f. Dehalobacteriaceae | 0.0002 | 0.0014 | -0.32 | 3.023 | NA |
|  | f. EtOH8 | 0.0007 | 0.0001 | -0.79 | 3.045 | NA |
|  | g. *Anaerofustis* | 0.0008 | 0.0003 | -1.29 | 3.024 | NA |
|  | g. *Pseudoramibacter* | 0.0076 | 0.0120 | 1.93 | 0.936 | 0.082 |
|  | g. *Anaerostipes* | 0.0083 | 0.0606 | 1.07 | 0.485 | 0.063 |
|  | g. *Lachnobacterium* | 0.0061 | 0.0079 | -1.10 | 1.299 | 0.529 |
|  | g. *Lachnospira* | 0.0006 | 0.0011 | -0.38 | 3.008 | NA |
|  | g. *Moryella* | 0.0026 | 0.0014 | -1.05 | 1.487 | 0.608 |
|  | g. *Oribacterium* | 0.0000 | 0.0003 | -0.44 | 3.039 | NA |
|  | g. *Shuttleworthia* | 0.0018 | 0.0022 | -0.24 | 0.747 | NA |
|  | f. Peptococcaceae | 0.0082 | 0.0089 | -0.49 | 0.679 | 0.608 |
|  | g. *Peptococcus* | 0.0140 | 0.0004 | -0.87 | 3.035 | 0.867 |
|  | g. *Anaerofilum* | 0.0004 | 0.0013 | 0.04 | 3.005 | NA |
|  | g. *Anaerotruncus* | 0.0035 | 0.0096 | 0.38 | 0.716 | 0.719 |
|  | f. Ruminococcaceae | 4.1190 | 4.7817 | -0.14 | 0.159 | 0.525 |
|  | g. *Oscillospira* | 1.2301 | 1.8552 | 0.31 | 0.154 | 0.090 |
|  | g. *Acidaminococcus* | 0.1706 | 0.0230 | -1.26 | 0.585 | 0.069 |
|  | g. *Dialister* | 0.0132 | 0.0113 | 0.00 | 0.483 | 0.992 |
|  | g. *Megamonas* | 0.1528 | 0.0085 | -0.73 | 0.767 | 0.479 |
|  | g. *Megasphaera* | 0.3364 | 0.1060 | -0.34 | 0.420 | 0.545 |
|  | g. *Mitsuokella* | 0.0119 | 0.0043 | -0.79 | 3.016 | 0.871 |
|  | g. *Phascolarctobacterium* | 0.2579 | 0.4401 | 0.38 | 0.219 | 0.151 |
|  | g. *Selenomonas* | 0.0000 | 0.0002 | -0.67 | 3.044 | NA |
|  | g. *Succiniclasticum* | 0.0007 | 0.0004 | -0.90 | 3.034 | NA |
|  | g. *vadinHB04* | 0.0000 | 0.0003 | -0.67 | 3.044 | NA |
|  | g. *Veillonella* | 0.0065 | 0.0003 | -1.53 | 3.017 | 0.728 |
|  | g. *Adlercreutzia* | 0.0036 | 0.0062 | -0.10 | 0.669 | 0.921 |
|  | g. *Atopobium* | 0.0033 | 0.0081 | -1.29 | 1.156 | 0.393 |
|  | f. Coriobacteriaceae | 1.6078 | 1.6172 | 0.27 | 0.310 | 0.517 |
|  | g. *Olsenella* | 0.0275 | 0.0411 | 0.74 | 0.424 | 0.148 |
|  | g. *Slackia* | 0.0003 | 0.0003 | -0.84 | 3.030 | NA |
|  | g. *Catenibacterium* | 0.2044 | 0.0361 | -0.96 | 0.793 | 0.339 |
|  | g. *Coprobacillus* | 0.0717 | 0.0794 | -0.91 | 0.443 | 0.082 |
|  | g. *Sharpea* | 0.4487 | 1.5755 | 0.04 | 0.357 | 0.946 |
|  | g. *Holdemania* | 0.0317 | 0.0380 | -0.11 | 0.325 | 0.837 |
|  | g. *L7A_E11* | 0.0004 | 0.0019 | -0.31 | 3.024 | NA |
|  | g. *p-75-a5* | 0.0091 | 0.0013 | -0.26 | 3.001 | 0.952 |
|  | o. Erysipelotrichales | 0.0780 | 0.0239 | -0.65 | 0.528 | 0.328 |
|  | o. NA | 0.1296 | 0.1485 | -0.32 | 0.198 | 0.188 |
|  | o. ML615J-28 | 0.0038 | 0.0991 | 0.82 | 0.853 | 0.479 |
| Fusobacteria | f. Fusobacteriaceae | 0.0523 | 0.0272 | -1.58 | 0.950 | 0.170 |
|  | o. Fusobacteriales | 0.0000 | 0.0001 | -0.73 | 3.048 | NA |
| Lentisphaerae | o. NA | 0.0002 | 0.0003 | -0.68 | 3.037 | NA |
|  | f. Victivallaceae | 0.0019 | 0.0066 | -0.71 | 2.991 | 0.877 |
| Proteobacteria | g. *Mycoplana* | 0.0001 | 0.0000 | -0.92 | 3.048 | NA |
|  | f. Caulobacteraceae | 0.0002 | 0.0005 | -0.51 | 3.028 | NA |
|  | g. *Phenylobacterium* | 0.0002 | 0.0000 | -0.86 | 3.048 | NA |
|  | o. NA | 0.0014 | 0.0032 | 0.62 | 2.981 | NA |
|  | f. Brucellaceae | 0.0004 | 0.0001 | -0.99 | 3.037 | NA |
|  | g. *Devosia* | 0.0000 | 0.0001 | -0.74 | 3.048 | NA |
|  | g. *Hyphomicrobium* | 0.0002 | 0.0001 | -0.85 | 3.045 | NA |
|  | o. Rhizobiales | 0.0007 | 0.0005 | -0.89 | 3.021 | NA |
|  | f. Phyllobacteriaceae | 0.0004 | 0.0002 | -1.04 | 3.038 | NA |
|  | g. *Paracoccus* | 0.0001 | 0.0000 | -0.90 | 3.048 | NA |
|  | g. *Rubellimicrobium* | 0.0000 | 0.0002 | -0.74 | 3.048 | NA |
|  | f. mitochondria | 0.0001 | 0.0001 | -0.81 | 3.048 | NA |
|  | f. Erythrobacteraceae | 0.0000 | 0.0002 | -0.77 | 3.048 | NA |
|  | o. Sphingomonadales | 0.0001 | 0.0000 | -0.82 | 3.048 | NA |
|  | g. *Sphingomonas* | 0.0000 | 0.0002 | -0.73 | 3.048 | NA |
|  | f. Alcaligenaceae | 0.0206 | 0.0036 | -1.01 | 1.083 | 0.484 |
|  | g. *Sutterella* | 0.5311 | 0.5241 | 0.02 | 0.275 | 0.952 |
|  | g. *Acidovorax* | 0.0003 | 0.0009 | -0.16 | 3.009 | NA |
|  | f. Comamonadaceae | 0.0171 | 0.0098 | -1.07 | 1.053 | 0.453 |
|  | g. *Rubrivivax* | 0.0002 | 0.0001 | -0.86 | 3.044 | NA |
|  | o. Burkholderiales | 0.0070 | 0.0082 | -0.18 | 0.412 | 0.772 |
|  | f. Oxalobacteraceae | 0.0006 | 0.0007 | -0.78 | 3.018 | NA |
|  | g. *Oxalobacter* | 0.0001 | 0.0002 | -0.75 | 3.042 | NA |
|  | f. Bacteriovoracaceae | 0.0000 | 0.0001 | -0.70 | 3.048 | NA |
|  | g. *Bilophila* | 0.0105 | 0.0148 | -0.90 | 0.661 | 0.268 |
|  | g. *Desulfovibrio* | 0.7747 | 0.7425 | -0.18 | 0.269 | 0.630 |
|  | f. Desulfovibrionaceae | 0.0057 | 0.0070 | 0.47 | 0.492 | 0.479 |
|  | o. Desulfovibrionales | 0.0002 | 0.0004 | -0.62 | 3.026 | NA |
|  | o. GMD14H09 | 0.0094 | 0.0153 | -0.91 | 3.012 | 0.860 |
|  | f. Haliangiaceae | 0.0003 | 0.0000 | -0.86 | 3.048 | NA |
|  | o. NA | 0.0003 | 0.0012 | 0.35 | 3.006 | NA |
|  | g. *Campylobacter* | 0.0086 | 0.0043 | -0.97 | 0.673 | 0.235 |
|  | f. Helicobacteraceae | 0.0001 | 0.0000 | -0.89 | 3.049 | NA |
|  | f. Aeromonadaceae | 0.0001 | 0.0001 | -0.77 | 3.046 | NA |
|  | o. Aeromonadales | 0.0006 | 0.0003 | -0.69 | 3.033 | NA |
|  | g. *Anaerobiospirillum* | 0.0002 | 0.0002 | -0.93 | 3.035 | NA |
|  | f. Succinivibrionaceae | 0.0040 | 0.0111 | 0.96 | 0.505 | 0.111 |
|  | g. *Ruminobacter* | 0.0330 | 0.0916 | 0.85 | 0.462 | 0.124 |
|  | g. *Shewanella* | 0.0005 | 0.0006 | -0.88 | 3.016 | NA |
|  | g. *Escherichia* | 1.2090 | 0.3857 | -0.61 | 0.376 | 0.182 |
|  | f. Enterobacteriaceae | 0.0527 | 0.0205 | -0.69 | 0.469 | 0.225 |
|  | o. NA | 0.0058 | 0.0041 | -0.42 | 0.743 | 0.696 |
|  | g. *Aggregatibacter* | 0.0001 | 0.0003 | -0.83 | 3.045 | NA |
|  | g. *Gallibacterium* | 0.0062 | 0.0008 | -1.48 | 2.991 | 0.729 |
|  | f. Pasteurellaceae | 0.0000 | 0.0001 | -0.73 | 3.048 | NA |
|  | g. *Acinetobacter* | 0.0006 | 0.0003 | -0.69 | 3.024 | NA |
|  | f. Pseudomonadaceae | 0.0007 | 0.0013 | -0.18 | 2.993 | NA |
|  | g. *Pseudomonas* | 0.0000 | 0.0002 | -0.63 | 3.044 | NA |
|  | f. Xanthomonadaceae | 0.0001 | 0.0000 | -0.92 | 3.049 | NA |
|  | o. NA | 0.0077 | 0.0140 | 0.52 | 0.382 | 0.271 |
| Spirochaetes | g. *Brachyspira* | 0.0000 | 0.0003 | -0.61 | 3.045 | NA |
|  | o. NA | 0.0000 | 0.0001 | -0.75 | 3.048 | NA |
|  | o. NA | 0.0002 | 0.0021 | 0.17 | 3.012 | NA |
|  | f. Spirochaetaceae | 0.0003 | 0.0033 | 0.70 | 3.005 | NA |
| Synergistetes | g. *Pyramidobacter* | 0.0010 | 0.0005 | -1.06 | 3.016 | NA |
|  | f. Synergistaceae | 0.0004 | 0.0000 | -1.01 | 3.045 | NA |
|  | g. *Synergistes* | 0.0001 | 0.0002 | -0.75 | 3.040 | NA |
| Tenericutes | g. *Anaeroplasma* | 0.0006 | 0.0797 | 1.56 | 2.973 | 0.719 |
|  | g. *RFN20* | 0.0045 | 0.0629 | 1.43 | 0.845 | 0.162 |
|  | g. *Mycoplasma* | 0.0003 | 0.0000 | -1.01 | 3.045 | NA |
|  | o. RF39 | 1.4395 | 1.2482 | -0.06 | 0.319 | 0.901 |
| Thermi | g. Meiothermus | 0.0000 | 0.0001 | -0.73 | 3.048 | NA |
| Verrucomicrobia | f. RFP12 | 0.0007 | 0.0000 | -0.89 | 3.048 | NA |
|  | g. *Akkermansia* | 0.0858 | 0.0405 | -0.54 | 0.756 | 0.608 |
|  | f. Verrucomicrobiaceae | 0.0000 | 0.0002 | -0.75 | 3.048 | NA |
| ^1^ Sequences that could not be assigned to a genus are displayed using the highest taxonomic level (class, c.; order, o.; family, f.) they could be assigned to. | | | | | | |
| ^2^ FC, fold change; SE, standard error for the log2 fold change estimate. | | | | | | |
| ^3^ If within a row, all samples have zero counts, the p-value will be "NA" | | | | | | |
