# Supplementary material for: Development of Ruminal and Fecal Microbiomes Are Affected by Weaning But Not Weaning Strategy in Dairy Calves
Source: Front Microbiol. 2016 May 3;7:582. doi: 10.3389/fmicb.2016.00582 (PMC4853645; doi:10.3389/fmicb.2016.00582)
Supplement: Supplementary file 3 [file Table_3.DOCX]

Supplementary Material

**Development of ruminal and fecal microbiomes are not affected by weaning strategy in dairy calves**

**S.J. Meale^1†‡^, S.C. Li^2†^, P. Azavedo^2^, H. Derakhshani^2^, J.C. Plaizier^2^, E. Khafipour^2,3,*^ and M.A. Steele^1,*^**

*** Co-corresponding authors:**

**M.A. Steele**, Department of Agricultural, Food and Nutritional Science, University of Alberta, Edmonton, AB, Canada.

**E. Khafipour**, Department of Animal Science; University of Manitoba, Winnipeg, MB, R3T 2N2, Canada; Tel: 204-474-6112; Fax: 204-474-7628; Email: Ehsan.Khafipour@umanitoba.ca

# Supplementary Tables

| **Table S3**. KEGG pathways with significantly different numbers of sequence hits in the rumen of pre- and post-weaned calves | | | | | |
| --- | --- | --- | --- | --- | --- |
| **KEGG Pathway level 3 (Class^1^)** | **Pre-wean** | **Post-wean** | **Log_2_ FC** | **Log_2_ SE** | **P-value** |
| Lysosome (C) | 0.16 | 0.09 | -0.79 | 0.135 | 0.000 |
| Peroxisome (C) | 0.22 | 0.16 | -0.42 | 0.063 | 0.000 |
| ABC transporters (E) | 2.27 | 2.82 | 0.38 | 0.071 | 0.000 |
| Phosphotransferase system (PTS) (E) | 0.38 | 0.71 | 0.99 | 0.307 | 0.009 |
| Secretion system (E) | 1.10 | 1.17 | 0.16 | 0.041 | 0.001 |
| Transporters (E) | 4.73 | 5.76 | 0.33 | 0.066 | 0.002 |
| Cellular antigens (E) | 0.08 | 0.05 | -0.63 | 0.144 | 0.007 |
| Protein processing in endoplasmic reticulum (G) | 0.09 | 0.07 | -0.24 | 0.049 | 0.000 |
| Sulfur relay system (G) | 0.18 | 0.22 | 0.39 | 0.089 | 0.000 |
| Base excision repair (G) | 0.45 | 0.45 | 0.07 | 0.018 | 0.010 |
| Chromosome (G) | 1.69 | 1.71 | 0.08 | 0.022 | 0.002 |
| DNA repair and recombination proteins (G) | 3.13 | 3.11 | 0.05 | 0.013 | 0.002 |
| DNA replication (G) | 0.76 | 0.75 | 0.04 | 0.014 | 0.016 |
| Homologous recombination (G) | 1.10 | 1.09 | 0.04 | 0.014 | 0.009 |
| Mismatch repair (G) | 0.90 | 0.89 | 0.04 | 0.018 | 0.031 |
| RNA polymerase (G) | 0.18 | 0.19 | 0.15 | 0.027 | 0.000 |
| Transcription factors (G) | 1.18 | 1.39 | 0.30 | 0.064 | 0.003 |
| Aminoacyl-tRNA biosynthesis (G) | 1.28 | 1.30 | 0.07 | 0.017 | 0.017 |
| Ribosome Biogenesis (G) | 1.52 | 1.59 | 0.13 | 0.021 | 0.000 |
| RNA transport (G) | 0.11 | 0.14 | 0.34 | 0.075 | 0.000 |
| Vibrio cholerae pathogenic cycle (H) | 0.09 | 0.10 | 0.28 | 0.068 | 0.002 |
| Arginine and proline metabolism (M) | 1.23 | 1.14 | -0.06 | 0.022 | 0.020 |
| Phenylalanine metabolism (M) | 0.20 | 0.18 | -0.16 | 0.046 | 0.005 |
| Phenylpropanoid biosynthesis (M) | 0.21 | 0.18 | -0.21 | 0.076 | 0.025 |
| Streptomycin biosynthesis (M) | 0.38 | 0.33 | -0.19 | 0.029 | 0.000 |
| Pentose phosphate pathway (M) | 0.82 | 0.92 | 0.20 | 0.043 | 0.005 |
| Propanoate metabolism (M) | 0.47 | 0.50 | 0.16 | 0.043 | 0.015 |
| Nitrogen metabolism (M) | 0.70 | 0.65 | -0.05 | 0.022 | 0.032 |
| Glycosaminoglycan degradation (M) | 0.12 | 0.09 | -0.47 | 0.168 | 0.013 |
| Glycosphingolipid biosynthesis - ganglio series (M) | 0.10 | 0.06 | -0.73 | 0.142 | 0.000 |
| Glycosphingolipid biosynthesis - globo series (M) | 0.16 | 0.10 | -0.59 | 0.114 | 0.000 |
| Other glycan degradation (M) | 0.39 | 0.24 | -0.64 | 0.092 | 0.000 |
| Peptidoglycan biosynthesis (M) | 0.93 | 0.93 | 0.06 | 0.022 | 0.029 |
| Biosynthesis of unsaturated fatty acids (M) | 0.11 | 0.12 | 0.20 | 0.046 | 0.002 |
| Fatty acid biosynthesis (M) | 0.48 | 0.51 | 0.15 | 0.026 | 0.000 |
| Glycerophospholipid metabolism (M) | 0.51 | 0.55 | 0.16 | 0.037 | 0.000 |
| Sphingolipid metabolism (M) | 0.23 | 0.15 | -0.64 | 0.101 | 0.000 |
| Pantothenate and CoA biosynthesis (M) | 0.69 | 0.63 | -0.08 | 0.025 | 0.011 |
| Porphyrin and chlorophyll metabolism (M) | 0.56 | 0.62 | 0.24 | 0.057 | 0.004 |
| Thiamine metabolism (M) | 0.47 | 0.50 | 0.14 | 0.033 | 0.000 |
| beta-Alanine metabolism (M) | 0.22 | 0.18 | -0.23 | 0.054 | 0.001 |
| D-Glutamine and D-glutamate metabolism (M) | 0.17 | 0.16 | -0.07 | 0.018 | 0.000 |
| Taurine and hypotaurine metabolism (M) | 0.13 | 0.11 | -0.20 | 0.041 | 0.001 |
| Biosynthesis of vancomycin group antibiotics (M) | 0.08 | 0.07 | -0.18 | 0.042 | 0.000 |
| Polyketide sugar unit biosynthesis (M) | 0.24 | 0.21 | -0.17 | 0.035 | 0.000 |
| Tetracycline biosynthesis (M) | 0.10 | 0.13 | 0.56 | 0.105 | 0.000 |
| Nitrotoluene degradation (M) | 0.03 | 0.04 | 0.51 | 0.139 | 0.002 |
| Adipocytokine signaling pathway (O) | 0.10 | 0.06 | -0.58 | 0.131 | 0.005 |
| PPAR signaling pathway (O) | 0.12 | 0.10 | -0.32 | 0.083 | 0.007 |
| Inorganic ion transport and metabolism (U) | 0.18 | 0.16 | -0.16 | 0.038 | 0.000 |
| Other transporters (U) | 0.25 | 0.27 | 0.17 | 0.036 | 0.000 |
| Signal transduction mechanisms (U) | 0.39 | 0.45 | 0.24 | 0.040 | 0.000 |
| Replication, recombination and repair proteins (U) | 0.67 | 0.81 | 0.34 | 0.050 | 0.000 |
| Restriction enzyme (U) | 0.19 | 0.17 | -0.12 | 0.037 | 0.004 |
| Translation proteins (U) | 0.99 | 1.01 | 0.10 | 0.016 | 0.000 |
| Amino acid metabolism (U) | 0.21 | 0.17 | -0.23 | 0.070 | 0.006 |
| Energy metabolism (U) | 1.04 | 0.91 | -0.15 | 0.030 | 0.000 |
| Metabolism of cofactors and vitamins (U) | 0.10 | 0.10 | 0.15 | 0.050 | 0.009 |
| Function unknown (U) | 1.28 | 1.31 | 0.09 | 0.030 | 0.010 |
| ^1^ C, Cellular Processes; E, Environmental Information Processing; G, Genetic Information Processing; M, Metabolism; O, Organismal Systems; U, Unclassified; H, Human Diseases; FC, fold change | | | | | |
