# Supplementary material for: Development of Ruminal and Fecal Microbiomes Are Affected by Weaning But Not Weaning Strategy in Dairy Calves
Source: Front Microbiol. 2016 May 3;7:582. doi: 10.3389/fmicb.2016.00582 (PMC4853645; doi:10.3389/fmicb.2016.00582)
Supplement: Supplementary file 4 [file Table_4.DOCX]

Supplementary Material

**Development of ruminal and fecal microbiomes are not affected by weaning strategy in dairy calves**

**S.J. Meale^1†‡^, S.C. Li^2†^, P. Azavedo^2^, H. Derakhshani^2^, J.C. Plaizier^2^, E. Khafipour^2,3,*^ and M.A. Steele^1,*^**

*** Co-corresponding authors:**

**M.A. Steele**, Department of Agricultural, Food and Nutritional Science, University of Alberta, Edmonton, AB, Canada.

**E. Khafipour**, Department of Animal Science; University of Manitoba, Winnipeg, MB, R3T 2N2, Canada; Tel: 204-474-6112; Fax: 204-474-7628; Email: Ehsan.Khafipour@umanitoba.ca

# Supplementary Tables

| **Table S4**. KEGG pathways with significantly different numbers of sequence hits in the feces of pre- and post-weaned calves | | | | | |
| --- | --- | --- | --- | --- | --- |
| **KEGG Pathway level 3 (Class^1^)** | **Pre-wean** | **Post-wean** | **Log_2_ FC** | **Log_2_ SE** | **P-value** |
| Bacterial chemotaxis (C) | 0.27 | 0.37 | 0.46 | 0.096 | 0.000 |
| Bacterial motility proteins (C) | 0.37 | 0.69 | 0.91 | 0.142 | 0.000 |
| Cytoskeleton proteins (C) | 0.40 | 0.37 | -0.13 | 0.030 | 0.000 |
| Flagellar assembly (C) | 0.12 | 0.27 | 1.17 | 0.177 | 0.000 |
| Peroxisome (C) | 0.17 | 0.19 | 0.17 | 0.052 | 0.013 |
| ABC transporters (E) | 3.31 | 3.00 | -0.17 | 0.055 | 0.025 |
| Secretion system (E) | 0.95 | 1.07 | 0.14 | 0.036 | 0.001 |
| Transporters (E) | 6.92 | 6.12 | -0.21 | 0.056 | 0.005 |
| RNA degradation (G) | 0.48 | 0.50 | 0.05 | 0.016 | 0.023 |
| Sulfur relay system (G) | 0.26 | 0.23 | -0.21 | 0.035 | 0.000 |
| Base excision repair (G) | 0.44 | 0.44 | -0.05 | 0.019 | 0.023 |
| Transcription factors (G) | 1.70 | 1.50 | -0.21 | 0.048 | 0.000 |
| RNA transport (G) | 0.16 | 0.14 | -0.20 | 0.055 | 0.001 |
| Translation factors (G) | 0.56 | 0.59 | 0.05 | 0.015 | 0.005 |
| Alanine, aspartate and glutamate metabolism (M) | 1.17 | 1.14 | -0.07 | 0.016 | 0.000 |
| Arginine and proline metabolism (M) | 1.28 | 1.23 | -0.09 | 0.020 | 0.000 |
| Glycine, serine and threonine metabolism (M) | 0.85 | 0.84 | -0.05 | 0.013 | 0.005 |
| Lysine biosynthesis (M) | 0.91 | 0.85 | -0.14 | 0.026 | 0.000 |
| Phenylalanine, tyrosine and tryptophan biosynthesis (M) | 0.90 | 0.87 | -0.08 | 0.023 | 0.003 |
| Tryptophan metabolism (M) | 0.11 | 0.13 | 0.17 | 0.041 | 0.001 |
| Tyrosine metabolism (M) | 0.33 | 0.33 | -0.08 | 0.025 | 0.010 |
| Valine, leucine and isoleucine biosynthesis (M) | 0.79 | 0.75 | -0.10 | 0.028 | 0.001 |
| Valine, leucine and isoleucine degradation (M) | 0.17 | 0.20 | 0.16 | 0.040 | 0.004 |
| Butanoate metabolism (M) | 0.57 | 0.61 | 0.06 | 0.023 | 0.033 |
| C5-Branched dibasic acid metabolism (M) | 0.33 | 0.31 | -0.12 | 0.040 | 0.007 |
| Citrate cycle (TCA cycle) (M) | 0.56 | 0.65 | 0.18 | 0.041 | 0.006 |
| Fructose and mannose metabolism (M) | 1.12 | 1.03 | -0.17 | 0.037 | 0.000 |
| Galactose metabolism (M) | 0.84 | 0.80 | -0.12 | 0.029 | 0.001 |
| Glyoxylate and dicarboxylate metabolism (M) | 0.52 | 0.50 | -0.10 | 0.019 | 0.000 |
| Inositol phosphate metabolism (M) | 0.10 | 0.10 | -0.10 | 0.041 | 0.043 |
| Pentose and glucuronate interconversions (M) | 0.57 | 0.53 | -0.12 | 0.034 | 0.003 |
| Pentose phosphate pathway (M) | 0.95 | 0.90 | -0.12 | 0.031 | 0.004 |
| Starch and sucrose metabolism (M) | 1.10 | 1.05 | -0.10 | 0.026 | 0.005 |
| Methane metabolism (M) | 1.42 | 1.34 | -0.11 | 0.025 | 0.000 |
| Photosynthesis (M) | 0.42 | 0.40 | -0.10 | 0.034 | 0.012 |
| Photosynthesis proteins (M) | 0.42 | 0.40 | -0.10 | 0.034 | 0.011 |
| Protein kinases (M) | 0.29 | 0.26 | -0.19 | 0.039 | 0.000 |
| Lipopolysaccharide biosynthesis (M) | 0.16 | 0.24 | 0.54 | 0.156 | 0.018 |
| Fatty acid biosynthesis (M) | 0.50 | 0.48 | -0.07 | 0.020 | 0.002 |
| Linoleic acid metabolism (M) | 0.09 | 0.08 | -0.21 | 0.036 | 0.000 |
| Lipid biosynthesis proteins (M) | 0.56 | 0.59 | 0.04 | 0.015 | 0.018 |
| One carbon pool by folate (M) | 0.62 | 0.68 | 0.10 | 0.016 | 0.000 |
| Porphyrin and chlorophyll metabolism (M) | 1.02 | 0.80 | -0.37 | 0.061 | 0.000 |
| Thiamine metabolism (M) | 0.53 | 0.51 | -0.08 | 0.021 | 0.001 |
| Vitamin B6 metabolism (M) | 0.19 | 0.21 | 0.12 | 0.030 | 0.001 |
| D-Glutamine and D-glutamate metabolism (M) | 0.15 | 0.16 | 0.07 | 0.018 | 0.002 |
| Taurine and hypotaurine metabolism (M) | 0.11 | 0.12 | 0.10 | 0.027 | 0.001 |
| Prenyltransferases (M) | 0.31 | 0.33 | 0.08 | 0.022 | 0.020 |
| Tetracycline biosynthesis (M) | 0.17 | 0.14 | -0.30 | 0.058 | 0.000 |
| Bisphenol degradation (M) | 0.10 | 0.09 | -0.23 | 0.040 | 0.000 |
| Chloroalkane and chloroalkene degradation (M) | 0.21 | 0.19 | -0.20 | 0.044 | 0.001 |
| Drug metabolism - other enzymes (M) | 0.31 | 0.33 | 0.06 | 0.017 | 0.009 |
| Nitrotoluene degradation (M) | 0.10 | 0.09 | -0.24 | 0.088 | 0.017 |
| Polycyclic aromatic hydrocarbon degradation (M) | 0.12 | 0.11 | -0.16 | 0.037 | 0.000 |
| Adipocytokine signaling pathway (O) | 0.06 | 0.07 | 0.32 | 0.111 | 0.009 |
| Glutamatergic synapse (O) | 0.12 | 0.11 | -0.12 | 0.028 | 0.000 |
| Cell motility and secretion (U) | 0.13 | 0.16 | 0.24 | 0.051 | 0.000 |
| Other ion-coupled transporters (U) | 1.23 | 1.16 | -0.11 | 0.023 | 0.000 |
| Signal transduction mechanisms (U) | 0.48 | 0.44 | -0.17 | 0.039 | 0.000 |
| Sporulation (U) | 0.82 | 0.69 | -0.27 | 0.093 | 0.022 |
| Carbohydrate metabolism (U) | 0.18 | 0.15 | -0.27 | 0.056 | 0.000 |
| Energy metabolism (U) | 0.86 | 0.95 | 0.12 | 0.027 | 0.002 |
| Others (U) | 0.89 | 0.85 | -0.10 | 0.032 | 0.014 |
| General function prediction only (U) | 3.71 | 3.67 | -0.05 | 0.012 | 0.002 |
| ^1^ C, Cellular Processes; E, Environmental Information Processing; G, Genetic Information Processing; M, Metabolism; O, Organismal Systems; U, Unclassified; FC, fold change | | | | | |
